# Essential genes shape cancer genomes through linear limitation of homozygous deletions

**DOI:** 10.1038/s42003-019-0517-0

**Published:** 2019-07-19

**Authors:** Maroulio Pertesi, Ludvig Ekdahl, Angelica Palm, Ellinor Johnsson, Linnea Järvstråt, Anna-Karin Wihlborg, Björn Nilsson

**Affiliations:** 1 Hematology and Transfusion Medicine Department of Laboratory Medicine, BMC, SE-221 84 Lund, Sweden; 2 Broad Institute, 415 Main Street, Cambridge, MA 02142 USA

**Keywords:** Lung cancer, Cancer genomics, Computational models

## Abstract

The landscape of somatic acquired deletions in cancer cells is shaped by positive and negative selection. Recurrent deletions typically target tumor suppressor, leading to positive selection. Simultaneously, loss of a nearby essential gene can lead to negative selection, and introduce latent vulnerabilities specific to cancer cells. Here we show that, under basic assumptions on positive and negative selection, deletion limitation gives rise to a statistical pattern where the frequency of homozygous deletions decreases approximately linearly between the deletion target gene and the nearest essential genes. Using DNA copy number data from 9,744 human cancer specimens, we demonstrate that linear deletion limitation exists and exposes deletion-limiting genes for seven known deletion targets (*CDKN2A*, *RB1, PTEN*, *MAP2K4*, *NF1*, *SMAD4*, and *LINC00290*). Downstream analysis of pooled CRISPR/Cas9 data provide further evidence of essentiality. Our results provide further insight into how the deletion landscape is shaped and identify potentially targetable vulnerabilities.

## Abstract (web summary)

Maroulio Pertesi, Ludvig Ekdahl et al. identify genes that limit homozygous deletions in cancer cells. This study provides insight into how the location of essential genes affects deletion events, which can be used to identify potentially targetable vulnerabilities in cancer.

## Introduction

Deletion of chromosomal material is a common feature of cancer genomes^1^. In addition to the target (driver) gene, these lesions often involve neighboring (passenger) genes^2,3^, some of which may be essential for the survival of tumor cells. While inactivation of the target gene contributes to cancer development, homozygous loss of a nearby essential gene will lead to clonal elimination, limiting the extent of chromosomal deletions. Additionally, hemizygous co-deletion of an essential gene can create a latent vulnerability in the tumor cells^4–13^, and a therapeutic window for drugs that further perturb the function of these genes or the processes in which they are involved^8–10,14^.

By now, the main sites of recurrent deletions have been identified. Yet, the essential genes that limit the extent of deletions at these loci have not been mapped, and the genomic patterns associated with deletion limitation have not been defined. The identification of essential genes currently relies on loss-of-function screens with shRNA/sgRNA libraries^7,15–20^. Here, however, we explored the possibility to identify flanking essential genes through their limiting effect on the extent of homozygous deletions. We argue that, under basic assumptions on positive and negative selection, the presence of essential genes near a deletion target gene gives rise to a statistical pattern where the frequency of homozygous deletions decreases linearly between the deletion target gene and the nearest essential genes. Using DNA copy number copy number data from 9744 cancer specimens belonging to 39 cancer subtypes, we show that linear deletion limitation exists, and exploit it to expose deletion-limiting genes for seven deletion targets (*CDKN2A*, *RB1, PTEN*, *MAP2K4*, *NF1*, *SMAD4*, and *LINC00290*). Subsequent analysis of CRISPR/Cas9 data further supports that the identified deletion-limiting genes are essential genes. Our results provide further insight into the anatomy of cancer genomes and identify potentially targetable vulnerabilities.

## Results

### Computational approach

One way to identify essential genes based on DNA CN data would be to select genes that are never homozygously deleted. Yet, this simple filter is unspecific, as large portions of the genome are never homozygously deleted. Instead, we developed a pattern-based method to identify essential genes by exploiting their limiting effect on the extent of homozygous deletions.

Basically, the landscape of somatic deletions is the result of a random process coupled to clonal selection. Deletion of specific target genes (e.g., tumor suppressors) is thought to lead to positive selection, and the deletion breakpoints vary randomly around these genes. However, in the case of homozygous deletions, the breakpoints can be expected to be located between the target gene and the nearest p- and q-terminal essential genes due to negative selection.

**Fig. 1:**
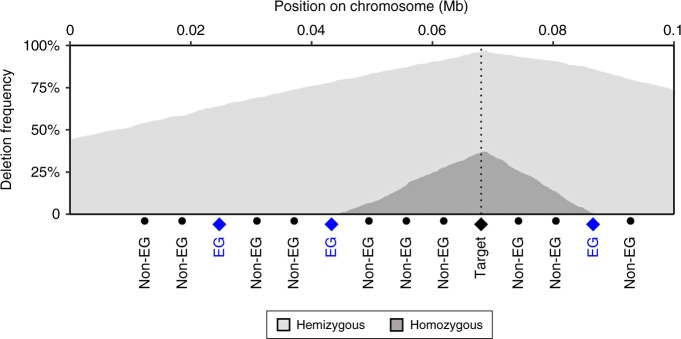
Linear deletion limitation illustrated by simulation. Hypothetically, the frequency of homozygous deletions in a recurrently deleted region should approach to zero at the nearest essential gene (EG). To illustrate this phenomenon, we simulated frequency of hemizygous and homozygous deletions across a fictive chromosomal region, harboring a deletion target gene surrounded by essential and non-essential genes. In these simulations, we required both copies of the target gene to be inactivated, however potentially through a variety of different mechanisms: regional deletion, whole-arm deletions, point mutation, or reduplication of the mutated chromosomes accompanied by deletion of the normal chromosome. The probability of each type of inactivation was determined by fixed probabilities. This plot shows the results across 1000 simulated chromosome pairs, and probabilities 20% for regional deletion, 7% for point mutation, and 1% for reduplication. Similar results were obtained with other parameter values. Regardless of parameter values, the frequency of homozygous deletions drops to zero at the essential genes located closer to the target gene

Now, if we assume that all breakpoints between the target gene and the nearest essential genes are equally probable, the positions of the p- and q-terminal breakpoints will be uniformly distributed in their respective intervals. As a result, the homozygous deletion frequency will decrease linearly from the target gene to the two nearest essential genes, as the cumulative distribution of a uniform density distribution is a straight line. This prediction contrasts with the naïve expectation that essential genes would create sharp borders in the deletion landscape. When the limiting essential gene and deletion target are close to each other and there are many deletion events, the slope of the decay line can become steep, to the point where it can be perceived as a sharp border. Technically, however, the homozygous deletion frequency will still decay gradually, and there will not be a sudden vertical drop (Heaviside step) at the essential gene. The phenomenon can be illustrated by simple simulation (Fig. 1 and Supplementary Fig. 1).

In this context, we recognize that the breakpoints may not always be perfectly uniformly distributed across the entire interval between the target gene and the limiting essential gene. For example, some positions could represent fragile sites with higher breakpoint probability. Nevertheless, it is a reasonable working assumption that the breakpoints are approximately uniformly distributed at least in some neighborhood close to the deletion-limiting essential gene, and that the homozygous deletion frequency decays approximately linearly in that neighborhood.

Based on this reasoning, we defined a deletion-limitation score (DLS) for a given DNA copy number dataset by first selecting genes that are conserved against homozygous deletions, then define the DLS as the linear correlation between homozygous deletion frequency and genomic position across a fixed-size neighborhood p-terminal or q-terminal of each of gene (i.e., the positive correlation coefficient for a straight line with zero intercept at the q-terminal end of the gene and raising across a q-terminal neighborhood; or the negative correlation coefficient of a straight line with zero intercept at the p-terminal end of the gene). The final score is given by the correlation coefficient with the largest absolute values. A DLS with a high absolute value thus means that the homozygous deletion frequency can be successfully modeled as a straight line, which is what we predict to happen theoretically in the vicinity of EGs. Consequently, if genes with high DLS can be detected, linear deletion limitation likely occurs near them.

When interpreting the DLS, one needs to bear in mind that the regression integrates information across a chromosomal neighborhood with a certain size. Thus, scores may be smoothed across genes with overlapping regression neighborhoods, and the size of the neighborhood determines the degree of smoothing. Using a smaller neighborhood will capture deletion-limiting phenomena at higher resolution (less smoothing), while producing scores based on fewer deletion events. Conversely, a larger neighborhood will produce scores based on more deletion events, while increasing smoothing.

To call homozygous (complete) deletions in order to identify deletion limitation, we applied dataset-specific thresholds to the copy number data. To select these thresholds, we recognize that we need to identify a set of lesions that are likely homozygous, with as little contamination from hemizygous deletions as possible, whereas it is not necessary to identify all homozygous deletions in an exhaustive manner. For this reason, we identified thresholds that call homozygous lesions conservatively (Supplementary Fig. 2). The thresholds were also justified using a theoretical model that accounts for variation in tumor cell fraction (see Methods and Supplementary Fig. 3) as well as the possibility that cancer genomes may contain more than two copies of the original chromosomal region.

### Identification of deletion-limiting genes

We applied our approach to pre-existing DNA copy number profiles of 7268 primary tumor samples from 24 tumor types from the Cancer Genome Atlas (TCGA)^21^. We calculated DLS using a 0.5-, 1- and 2-Mb neighborhood, selected genes with DLS greater than 0.8 based on at least 15 deletion events and assessed the robustness of the scores by bootstrapping. To focus our analysis on genes that have a potentially targetable vulnerability, we limited our analysis to genes with a reasonable (>5%) frequency of hemizygous deletions.

**Fig. 2:**
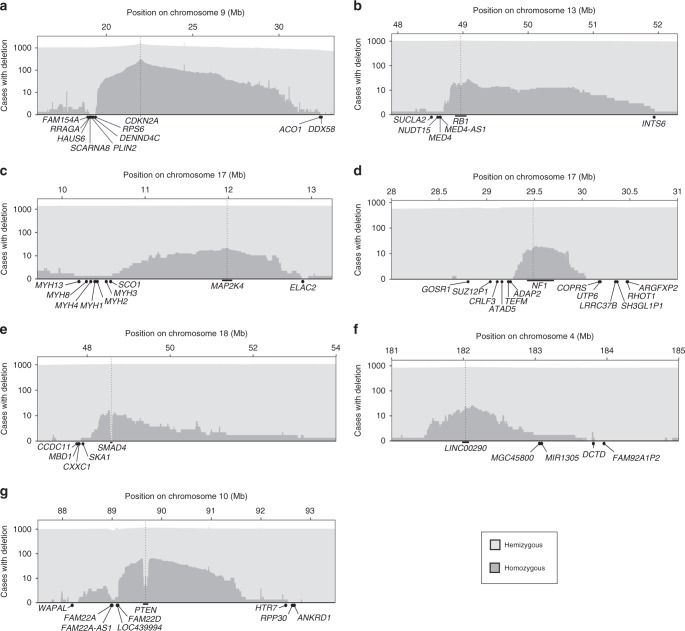
Computationally identified genomic regions harboring deletion-limiting genes. To test whether linear deletion limitation phenomenon exist in human tumors, we developed a score (DLS that captures the correlation between homozygous deletion frequency and genomic position along a straight line that extends across a neighborhood of a fixed size from either the annotated gene start or gene end. Applying this method to DNA copy number profiles of 7,268 tumor samples from the Cancer Genome Atlas (TCGA), we identified deletion limiting gene near seven deletion targets: *CDKN2A* (**a)**, *RB1* (**b**), *MAP2K4* (**bc**), *NF1* (**d**), *SMAD4* (**e**), *LINC00290* (**f**) and *PTEN* (**g**). The positions of the deletion-limiting genes identified in each region are indicated. At all seven loci, the homozygous deletion frequency decays to zero between the deletion target gene towards and the limiting genes in an approximately linear manner. For *SMAD4* and *PTEN*, we noted an unusual deletion pattern of homozygous deletions that appears to spare the target genes themselves. The reason for the latter is unclear, and could reflect a technical artifact (e.g., superposition of signal from non-deleted DNA with sequence homology with *SMAD4* or *PTEN*)

Using these criteria, we detected deletion limitation at 48 genes in seven distinct genomic regions (Table 1). For six of these, the deletion peaks map to well-known tumor suppressor genes (*CDKN2A*, *PTEN*, *RB1*, *MAP2K4*, *NF1*, and *SMAD4*) that are frequently deleted in multiple cancer types (Supplementary Fig. 4). For the seventh region, the deletion peak maps to a long intergenic non-coding RNA of putative cancer relevance (*LINC00290*)^22^. The candidate deletion-limiting genes were located p-terminal and/or q-terminal of their respective target genes, adjacent to the points where the homozygous deletion frequency approaches zero (Fig. 2a–g). For further validation, and to exclude that the results were due to admixture of non-tumor cells, we examined the seven loci in DNA copy number profiles of 1043 cancer cell lines from the Cancer Cell Line Encyclopedia (CCLE)^23^. In all seven regions, we observed deletion limitation patterns and DLS scores (Supplementary Fig. 5a–g, Supplementary Table 1) analogous to those observed in TCGA. Additionally, in a second set of DNA copy number profiles of 2476 tumor specimens from 39 tumor types (Tumorscape^24^), we observed deletion limitation in the *CDKN2A*, *MAP2K4*, and *PTEN* regions (Supplementary Fig. 6a–g) whereas the results were inconclusive in the other four regions, most likely because the latter data set was generated using lower-resolution microarrays (Affymetrix 250k) and lower-purity samples (median tumor cell fraction 58% compared to 89% and 99% for TCGA and CCLE; Supplementary Fig. 3), making it harder to detect homozygous deletions.

**Table 1:** Genes with high DLS scores in the TCGA dataset

GeneSymbol	Chr	Start	End	0.5 Mb			1 Mb			2 Mb		
				*r*	90% CI	*N*	*r*	90% CI	*N*	*r*	90% CI	*N*
4q34.3 *LINC00290*												
* LOC90768*^a^	4	183,060,158	183,065,668				−0.976	[−0.988 to −0.921]	25			
* MIR1305*^a^	4	183,090,445	183,090,531				−0.971	[−0.988 to −0.911]	25			
* DCTD*	4	183,811,243	183,838,630							−0.924	[−0.958 to −0.839]	25
* FAM92A1P2*^a^	4	183,958,817	183,961,272							−0.906	[−0.956 to −0.81]	25
9p21.3 *CDKN2A*												
* FAM154A*	9	18,927,890	19,033,256				0.932	[0.893 to 0.951]	40	0.991	[0.983 to 0.993]	86
* RRAGA*	9	19,049,371	19,051,021				0.944	[0.913 to 0.959]	41	0.992	[0.984 to 0.994]	93
* HAUS6*	9	19,053,134	19,102,940				0.962	[0.936 to 0.972]	41	0.993	[0.985 to 0.995]	102
* SCARNA8*^a^	9	19,063,653	19,063,784				0.949	[0.919 to 0.962]	41			
* PLIN2*	9	19,115,758	19,127,604				0.968	[0.945 to 0.977]	42	0.993	[0.984 to 0.995]	109
* DENND4C*	9	19,230,762	19,374,137	0.993	[0.971 to 0.995]	29	0.996	[0.985 to 0.997]	50	0.989	[0.975 to 0.995]	141
* RPS6*	9	19,376,253	19,380,235	0.997	[0.983 to 0.996]	32	0.996	[0.986 to 0.997]	50	0.988	[0.973 to 0.994]	141
* ACO1*	9	32,384,600	32,450,832							−0.919	[−0.944 to −0.716]	17
* DDX58*	9	32,455,299	32,526,322							−0.910	[−0.943 to −0.682]	17
10q23.31 *PTEN*												
* WAPAL*	10	88,195,012	88,281,541							0.913	[0.893 to 0.923]	63
* FAM22A*	10	88,985,204	88,994,733	0.973	[0.944 to 0.984]	38	0.906	[0.883 to 0.922]	59			
* FAM22A-AS1*^a^	10	88,998,423	89,102,315	0.995	[0.984 to 0.995]	58	0.913	[0.884 to 0.931]	62			
* LOC439994*	10	89,102,167	89103,331	0.994	[0.981 to 0.995]	56	0.913	[0.885 to 0.929]	62			
* FAM22D*	10	89,117,476	89,130,452	0.965	[0.951 to 0.968]	56	0.911	[0.881 to 0.929]	63			
* HTR7*	10	92,500,575	92,617,671							−0.933	[−0.959 to −0.867]	33
* RPP30*	10	92,631,708	92,668,312							−0.915	[−0.946 to −0.832]	28
* ANKRD1*	10	92,671,856	92,681,032							−0.908	[−0.941 to −0.819]	28
13q14.2 *RB1*												
* SUCLA2*	13	48,516,790	48,575,462	0.962	[0.908 to 0.972]	25						
* NUDT15*	13	48,611,702	48,621,282	0.975	[0.922 to 0.982]	26						
* MED4*	13	48,649,863	48,669,277	0.971	[0.916 to 0.979]	26						
* MED4-AS1*^a^	13	48,651,272	48,654,129	0.975	[0.924 to 0.982]	26						
17p12 *MAP2K4*												
* MYH13*	17	10,204,182	10,276,322							0.977	[0.933 to 0.982]	20
* MYH8*	17	10,293,641	10,325,267							0.968	[0.924 to 0.977]	20
* MYH4*	17	10,346,607	10,372,876							0.960	[0.909 to 0.973]	20
* MYH1*	17	10,395,626	10,421,859							0.950	[0.894 to 0.969]	20
* MYH2*	17	10,424,464	10,452,940							0.943	[0.883 to 0.966]	20
* MYH3*	17	10,531,842	10,560,626							0.912	[0.838 to 0.946]	20
* SCO1*	17	10,583,648	10,600,885							0.901	[0.825 to 0.94]	20
* ELAC2*	17	12894928	12921381				−0.989	[−0.991 to −0.946]	19			
17q11.2 *NF1*												
* GOSR1*	17	28,804,425	28,853,832				0.901	[0.824 to 0.923]	18			
* SUZ12P1*	17	29,036,625	29,097,068	0.951	[0.897 to 0.961]	18						
* CRLF3*	17	29,109,701	29,151,778	0.974	[0.928 to 0.981]	18						
* ATAD5*	17	29,159,022	29,222,295	0.964	[0.893 to 0.983]	17						
* TEFM*	17	29,226,000	29,233,286	0.959	[0.899 to 0.982]	18						
* ADAP2*	17	29,248,753	29,286,211	0.910	[0.823 to 0.953]	18						
* COPRS*	17	30,178,883	30,186,326	−0.951	[−0.975 to −0.854]	15						
* LRRC37B*	17	30,348,154	30,380,519				−0.925	[−0.946 to −0.852]	18			
* SH3GL1P1*^a^	17	30,367,354	30,369,851				−0.932	[−0.95 to −0.867]	18			
* RHOT1*	17	30,469,472	30,552,746				−0.916	[−0.933 to −0.856]	18			
* ARGFXP2*^a^	17	30,477,386	30,478,590				−0.913	[−0.931 to −0.851]	18			
18q21.2 *SMAD4*												
* CCDC11*	18	47,753,562	47,792,865				0.914	[0.843 to 0.925]	15			
* MBD1*	18	47,793,251	47,808,144				0.916	[0.841 to 0.928]	15			
* CXXC1*	18	47,808,712	47,814,692				0.916	[0.841 to 0.928]	15			
* SKA1*	18	47,901,391	47,920,538				0.920	[0.838 to 0.942]	15			

Candidate deletion-limiting genes show a DLS > 0.8 (*r*) within a 0.5-, 1- and/or 2-Mb neighborhood based on at least 15 homozygous somatic deletion events within the neighborhood (*N*), as well as >5% frequency of hemizygous deletions. Positive and negative *r*-values indicate candidate genes at the p- and q-terminal, res*p*ectively. To estimate the robustness of DLS scores, we assigned 90% confidence intervals using 200-fold bootstrapping. The identified genes map to seven distinct genomic regions, characterized by a high frequency of somatic acquired deletions with well-known deletion targets

^a^Non protein coding gene

Since the DLS reflects how well homozygous deletion frequency is explained locally by a linear model whose intercept with the zero baseline, these results indicate that linear deletion limitation occurs in human cancer, at least at these seven loci.

### Deletion-limiting genes at the identified loci

The genes with the highest DLS were located around *CDKN2A* on chromosome 9p21 (Fig. 2a and Table 1). Here, we found that the frequency of homozygous deletions drops quickly, and almost perfectly linearly, at *RPS6*. This observation, along with the fact that *RPS6* encodes one of the proteins of the small (40S) ribosomal subunit, indicates that *RPS6* is an essential gene and limits the p-terminal extent of regional, homozygous deletions targeting *CDKN2A*. Interestingly, *RPS6* also marks the q-terminal boundary of a 400-kb region that is never homozygously deleted, and thus likely harbors additional essential genes. On the q-terminal side, the frequency of homozygous deletions reached zero around *ACO1*. However, in contrast to the p-terminal side, where the homozygous deletion frequency drops to zero within a short distance of 2.5 Mb from *CDKN2A* and it is easy to identify *RPS6* as a likely limiting gene, the homozygous deletion frequency decays across a broader region of 10.4 Mb on the q-terminal side, making it more difficult to pinpoint the limiting gene based on the copy number distribution (as there will be fewer deletion events within the regression window). On the q-terminal side, *ACO1* thus marks the start of a region that is never homozygously deleted and harbors several likely essential genes such as *SMU1*, encoding a DNA replication regulator and spliceosomal factor^25^, and *NOL6*, encoding a protein required for ribosome biogenesis^26^.

The second strongest DLS signals were found around *RB1* at chromosome 13q14. Here, we predicted *MED4* as limiting gene on the p-terminal side (Fig. 2b, Table 1). This gene encodes a core subunit of the mediator complex that links transcription factor binding to the RNA polymerase II machinery^27^, and has previously been reported as a limiting gene for *RB1* deletions^28^. While no genes on the q-terminal side fulfilled our requirement for 15 homozygous deletion events, we noted that the homozygous deletion frequency dropped at *INTS6* (Fig. 2b**)**, which encodes one of the components of the integrator complex involved in transcription^27^ (DLS 0.98 with a 2.0 Mb neighborhood based on 12 deletion events).

Further, at *MAP2K4*, we identified the mitochondrial genes *SCO1* and *ELAC2*, involved in mtRNA processing^29^ and the cytochrome *c* complex^30^, respectively, as deletion-limiting (Fig. 2c). At *NF1*, we identified *TEFM*, required for mitochondrial transcription elongation^31^, *ADAP2* which binds beta-tubulin and increases the stability of microtubules^32^, and *COPRS*, a putative oncogene^33^ (Fig. 2d). At *SMAD4*, we identified *CXXC1*, encoding for a DNA methylation regulating CpG-binding protein, and *SKA1*, involved in mitotic spindle and kinetochore assembly, as the p-terminal deletion-limiting genes. Interestingly, downregulation of this gene has been reported to lead to reduced cell proliferation and invasiveness in cancer^34^ (Fig. 2e). At *LINC00290*, we identified *DCTD*, which encodes a dCMP deaminase required for nucleotide synthesis^35^, as the q-terminal deletion-limiting gene, while the p-terminal limiting point mapped to a gene desert (Fig. 2f). Lastly, at *PTEN*, which shows an unusual deletion distribution, the candidate deletion-limiting genes were *WAPAL*, encoding a cohesin-associated protein^36^, and *FAM22A* on the p-terminal side and *RPP30*, encoding a part of the RNase P complex^37^, on the q-terminal side (Fig. 2g).

Altogether, our results identify the limit points for homozygous deletions for several well-established tumor suppressor genes. The fact that the genes located at these points are involved in key metabolic processes (ribosomes, mitochondria, and transcription) supports that the deletion-limiting genes are essential for cell survival.

### Analysis of essentiality in the 9p21/*CDKN2A* region

**Fig. 3:**
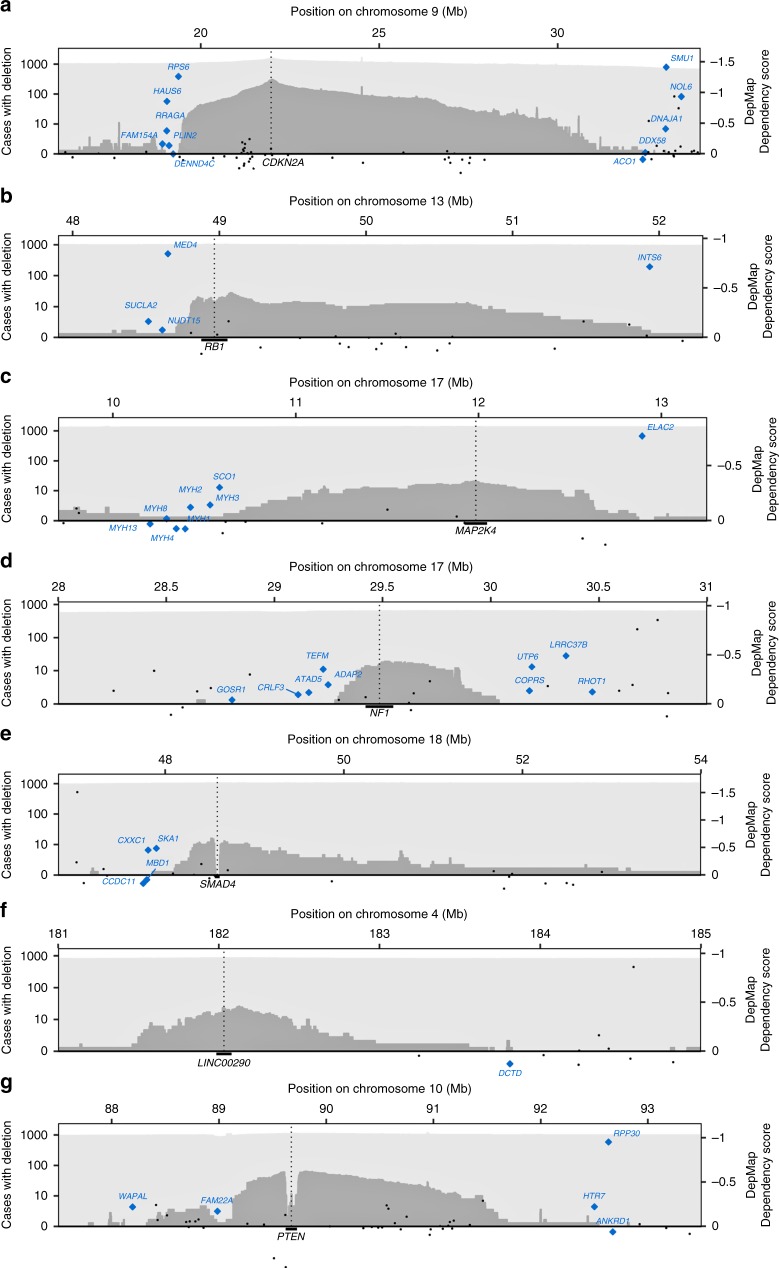
Correspondence of gene dependency scores with deletion patterns. This figure shows the gene dependency scores from pooled CRISPR-Cas9 screens for 558 cancer cell lines from the Cancer Dependency Map (DepMap AVANA 19Q1) for all genes located in the seven genomic regions where deletion-limitation was detected using copy number data: *CDKN2A* (**a**), *RB1* (**b**), *MAP2K4* (**c**), *NF1* (**d**), *SMAD4* (**e**), *LINC00290* (**f**) and *PTEN* (**g**). As shown, most of the computationally predicted that were located closest to the deletion targets showed depletion, albeit with varying effect sizes. Genes from Table 1 and Supplementary Table 2 indicated in blue. Remaining genes indicated in black

To explore further the essentiality of deletion-limiting genes at all seven loci, we first examined genome-wide CRISPR loss-of-function screen data from 558 cell lines from the Cancer Dependency Map^20,38^. Among the computationally identified deletion-limiting genes, *HAUS6* and *RPS6* (near *CDKN2A*), *MED4* and *INTS6* (near *RB1*), *ELAC2* (near *MAP2K4*), *WAPAL, FAM22A* and *RPP30* (near *PTEN*), *CXXC1* and *SKA1* (near *SMAD4*), and *TEFM*, *ADAP2*, and *COPRS* (near *NF1*) showed depletion by CRISPR-Cas9 in these data, further supporting essentiality (Fig. 3a–g, Supplementary Table 2 and http://www.depmap.org). We also noted that *SMU1*, *NOL6*, *RPS6*, and *WAPAL* are completely conserved against germline loss-of-function mutations in the GNOMAD database^39^ (pLI > 0.98), and *TEFM* and *COPRS* partly conserved (pLI = 0.51 and 0.25, respectively), though it is not given that a gene that is conserved against heterozygous loss-of-function variants in the germline (which is mainly what the pLI score reflects) is essential for the survival of tumor cells, or vice versa.

The strongest evidence for deletion-limitation was observed in the *CDKN2A* region. Because of the prominence of *CDKN2A* as a deletion target, and because the limiting gene on the q-terminal side was not clearly identified, we also carried out a focused CRISPR/Cas9 negative selection screen targeting a set of 68 genes flanking *CDKN2A*. The screened region ranged from 16.5 (*BNC2*) to 34 Mb (*UBAP2*), targeted by 398 sgRNA sequences from the human GeCKO v2.0 library^40^ (Supplementary Table 3). The resulting pooled sgRNA library was transduced into the human acute lymphoblastic leukemia (ALL) Jurkat and RCH-ACV. The representation of each sgRNA was assessed by deep sequencing of integrated sgRNA sequences at day 14 and compared to the representation at day 3 after puromycin selection. Gene depletion was quantified as the log_2_ fold change of each sgRNA. We considered genes for which at least two sgRNAs showed greater than 25% depletion as potential essential genes. We observed depletion of *HAUS6*, *RPS6*, *SMU1*, and *NOL6* in both cell lines, and selective depletion of *RRAGA* in RCH-ACV cells and *DNAJA1* in Jurkat cells (Supplementary Figs. 7a–c,  8a–c).

Both *RPS6* and *NOL6* genes are involved in ribosome biogenesis^26^. *RPS6* knockdown impairs ribosome biogenesis, activates p53^26^, and alters rRNA processing^41^. Knockdown of *nol6* in *C. elegans* disrupts nucleolar integrity and induces p53^26^. SMU1 is a chromatin-bound protein involved in the regulation of DNA replication^25^. HAUS6 is a key component of the HAUS-Augmin complex required for recruiting γ-tubulin to mitotic spindle microtubules^42^. Knockdown of both of these genes has been reported to result in cell cycle arrest and cell death^25,42^. As for the genes showing cell-line specific essentiality, *RRAGA* encodes a regulator of mTORC1 and knockdown leads to enhanced p53 translation and p53-dependent senescence^43^ via the PI3K/AKT pathway^44^. Thus, *RRAGA* knock-down could selectively affect the survival of cells with intact *TP53*, including RCH-ACV. On the other hand, *DNAJA1* binds mutant p53 and prevents its degradation^45^, and shows depletion only in Jurkat cells, which carry an inactivating *TP53* mutation (p.Arg196*)^46^. Thus, the different results for *RRAGA* and *DNAJA1*, and the more modest effect of *RPS6* and *NOL6* knockout seen in Jurkat could be explained by differences in *TP53* status.

In all, these observations further support that the identified deletion-limiting genes are essential and identify additional essential genes in the *CDKN2A* region.

### Exploiting haploinsufficiency of identified essential genes

While conserved for homozygous deletions, the identified essential genes are regularly co-deleted with their respective driver genes (Fig. 3a–g). For all identified essential genes except *FAM22A*, we observed a correlation between DNA copy number and transcript level across 947 human cancer cell lines from the CCLE^23^ (Supplementary Fig. 9), and data from pooled CRISPR/Cas9^20,38^ and shRNA knockdown screens^2,5,47^ indicate that hemizygous deletion of *HAUS6*, *RPS6*, *SMU1*, *NOL6, RPP30, MED4, INTS6*, and *ELAC2* increases the sensitivity to further knockdown of these genes (Supplementary Table 2). These data suggest that the haploinsufficiency for at least a subset of the identified essential genes could potentially be exploited for selective targeting. The identification of concrete ways to exploit haploinsufficiency for selective targeting is however beyond the scope of this study.

## Discussion

We have carried out a systematic analysis to identify genes that limit the extent of homozygous deletions in cancer genomes. Identifying genes that are essential for the survival of cancer cells (not to be confused with genes that are essential for cancer initiation) is potentially clinically relevant as they tend to be located close to key deletion target genes and tend to be frequently hemizygously deleted. It is conceivable that hemizygous loss (haploinsufficiency) of an essential gene creates a cancer cell-specific vulnerability in the form of a therapeutic window for drugs that further perturb the function of the essential gene.

In this study, we consider the possibility of identifying essential genes from DNA copy number data. We predict theoretically that, under basic assumptions of deletions arising through a random process coupled to both positive and negative selection, there should exist a statistical pattern where the frequency of homozygous deletions decays approximately linearly between the deletion target gene and the nearest essential gene. Using DNA CN data from 9744 cancer specimens, we demonstrate that linear limitation occurs in human cancer. This is evidenced by the detection of seven loci harboring genes with high DLS, which reflects how well homozygous deletion frequency is explained by a linear model with zero baseline across a neighborhood upstream or downstream of the limiting essential gene. For validation, we carry out replication analyses in CCLE, with good results, and in Tumorscape, where some patterns replicate but not all, probably due to lower resolution and lower tumor cell fraction. The reason for using microarray data, as opposed to for example whole-exome sequencing (WES) data, is that it provides dense copy number data across the entire genome, not just coding regions. This allows identification of linear relationships between homozygous deletion frequency and genome position. With WES data, one would only have deletion frequencies in coding regions (about 2–3% of the genome), which would be too sparse for this type of analysis.

The clearest example of linear deletion limitation was found at the *CDKN2A* region at 9p21, which is commonly deleted in several cancer types, including ALL, lymphoma, glioblastoma, and melanoma^24^. Here, we found that homozygous *CDKN2A* deletions are constrained by *RPS6* and identify *HAUS6* and *RRAGA* as additional essential genes in the p-terminal conserved region. On the q-terminal side, we identify *SMU1*, *NOL6*, and possibly *DNAJA1*, as likely essential genes. Both *RPS6* and *SMU1*, as well as several other predicted deletion-limiting genes are involved in key metabolic processes (e.g., ribosomes, cell division, mitochondria). Candidate limiting essential genes were also identified in the other regions, including *WAPAL*, *FAM22A*, and *RPP30* (near *PTEN*), *MED4* and *INTS6* (near *RB1*), *SCO1* and *ELAC2* (near MAP2K4), *CXXC1* and *SKA1* (near *SMAD4*), and *TEFM*, *ADAP2*, and *COPRS* (near *NF1*), all of which supported both by high deletion limitation scores and by varying degrees of depletion in shRNA and/or CRISPR-Cas9 screens.

At some of the detected loci, we noted that the homozygous deletion frequency appears to reach zero before the likely limiting essential gene. A likely explanation for this is that the number of deletion events in the available data sets is small. Theoretically, when the number of deletions with end points between the deletion target and the limiting essential gene is finite, one of them must be closest to the essential gene. Yet, this closest deletion does not have go all the way up to the essential gene. It can end before. Moreover, since the end points are randomly distributed between the deletion target and the limiting essential gene, the distance between the essential gene and closest end point will likely be larger when there are fewer deletions in the data set (i.e., data are locally sparse). Thus, for a gene to be a limiting essential gene, it is not correct to require that there exists an individual deletion that goes all the way up to that gene in a given data set. This criterion is only correct when the number of available deletion events is infinite, or at least very high.

A possible advantage of identifying essential genes via deletion frequencies is that this approach exposes genes that are essential under in vivo conditions, thereby avoiding the risk of detecting genes that are essential only to cancer cells in culture. Its main limitation is that it requires sufficient numbers of deletion events locally and that it smooths information across genes within the neighborhood used to calculate the DLS. Other limitations, which this approach shares with in vitro screens, are that it is not guaranteed to identify genes that are essential only to cancer cells and it is unclear to what extent it only detect genes that are fully essential or also genes that somewhat reduce fitness. Further, our model obviously has certain theoretical limitations in that it assumes that deletions are continuous, and the deletion breakpoints are approximately uniformly distributed in some neighborhood around the limiting essential gene. While these are appropriate working assumptions, and our approach appears to give reasonable results in practice, we firstly recognize that not all deletions are continuous. In some cases, the underlying lesion is more complex, involving for example chromothripsis that could generate lesions that are punctuated rather than continuous. Secondly, we also note that the breakpoint probability could be influenced by other factors, including fragile sites. Developing refined statistical models represents an interesting challenge ahead.

As an alternative to our model (which is based on random deletion and negative selection), a linear or near-linear trend could be consistent with a localized process. For example, if deletion lengths were distributed around some mean length related to the structure of that chromosomal region, then a similar decay could be observed in the absence of a limiting essential gene. While it is impossible to exclude this alternative model completely, it appears less likely as it would generate decay patterns not only for homozygous deletions, but also for hemizygous deletions at the same loci, which we do not see in Fig. 3, Supplementary Figs. 5 and 6.

An interesting question is how identified essential genes can be exploited therapeutically. Here we observe that all the identified limiting essential genes are also frequently hemizygously deleted (Fig. 3 and Supplementary Table 4), and most of them show copy number-dependent expression (Supplementary Fig. 9). This suggests that a substantial proportion of human cancers could have a therapeutic window for drugs that further perturb the function of the identified essential genes or the metabolic processes in which they are involved, with limited impact on non-tumor cells. The next challenge is to identify concrete ways to achieve this. In all, our results provide further insight into the anatomy of cancer genomes and identify potentially targetable vulnerabilities.

## Methods

### Cancer genome data sets

We obtained segmented copy number data for 7268 primary tumor samples belonging to 24 cancer types from The Cancer Genome Atlas (TCGA) (http://cancergenome.nih.gov/)^21^, 2476 samples belonging to 39 cancer types from the Tumorscape compendium (http://www.broadinstitute.org/tumorscape)^24^, and 1043 samples representing human cancer cell lines from the CCLE (https://portals.broadinstitute.org/ccle)^23^.

### Deletion calling

To identify deletion-limitation, we need to identify a set of homozygous (complete) deletions, with as little contamination from hemizygous (incomplete) deletions as possible. However, there is no need to find all complete deletions. Missing some of them may reduce sensitivity, as there will be fewer data points, but will not create false positives. Thus, what we need is copy number thresholds that allow us to call homozygous deletions conservatively.

To find appropriate thresholds, we need to know the distribution of copy numbers for hemizygous deletions. To learn these distributions, one can utilize the fact that very large deletions (i.e., those encompassing whole chromosomes or big parts of chromosomes) are almost always hemizygous, as the probability that a very large deletion will cover at least one essential gene is close to 1. Consequently, we can learn the distribution of CNs for hemizygous/incomplete deletions by looking at the CNs of deletions larger than in the order of 50–100 million base pairs. Using this type of analysis, we identified CN < 0.5 (corresponding to linear depth <−1.5 or log_2_ ratio <−2.0) as an appropriate threshold for TCGA, <0.75 (corresponding to linear depth <−1.25 or log_2_ ratio <−1.4) for Tumorscape, and <0.25 (corresponding to linear depth <−1.75 or log_2_ ratio <−3.0) for CCLE (Supplementary Fig. 2). We also repeated our experiments with other reasonable thresholds, yielding results in broad agreement with those presented.

The thresholds can also be motivated through theoretical calculations. Assuming well-normalized microarray data, the deletion depth observed on DNA copy number microarrays approximately equals the average deletion depth in the sample multiplied by a constant *k* that defines the scale of the copy number signal in linear scale (not in log_2_ scale). Ideally, *k* = 1 scale units/copy, but in practice *k* is slightly lower (empirically somewhere in the order of 0.95 scale units/copy for deletions on Affymetrix 6.0 arrays) as deletion depth is calculated by averaging the signal for several microarray probes and not all probes are efficient. So, assuming 100% tumor cell fraction, a homozygous deletion should show linear depth −2*k*, while a hemizygous deletion should show linear depth -1*k*. If the tumor cell fraction *t* is <100%, the deletion depth will also be proportional to *t*. Thus, one simple model is:

$${\mathrm{Observed}}\,{\mathrm{deletion}}\,{\mathrm{depth}} \cong k \cdot t \cdot {\mathrm{deletion}}\,{\mathrm{depth}}\,{\mathrm{in}}\,{\mathrm{tumor}}$$

To gain further insight into whether our choice of thresholds is appropriate, we thus estimated t per every sample in the TCGA, Tumorscape, and CCLE data sets. For this, we used an optimization-based approach. For a given candidate *t*, we defined hypothetical copy number centroids $k \cdot t \cdot d,d = - 2, - 1,0$ and assigned each segment *s* = 1,…, *S* for the sample to its closest hypothetical copy number centroid $k \cdot t \cdot d_{\mathrm{closest}}(s)$ based on its observed deletion depth d_s_. We then estimated the tumor cell fraction in the sample by minimizing the penalty function $\max \left| {k \cdot t \cdot d_{\mathrm{closest}}\left( s \right) - d_s} \right| \cdot {\mathrm{length}}\left( s \right){\mathrm{over}}\,0 \le t \le 1$. As shown in Supplementary Fig. 3, this yielded a median tumor cell fraction of 89% for TCGA, 58% for Tumorscape, and 99% for CCLE, which is consistent with the fact that CCLE represents cell lines, that TCGA represents tumor samples with >80% tumor cells microscopically, and that no tumor purity criterion was used when Tumorscape was generated. We note that log_2_(0.58/0.89) = −0.62 which is on par with the difference between the copy number thresholds used for TCGA and Tumorscape we inferred from CN distributions in Supplementary Fig. 2 (log_2_ ratio −2.0 vs −1.4).

Finally, for completeness, we note that the deletion depth could be influenced by the normalization of the microarray data. A basic assumption in the normalization is that the sample is euploid on average (i.e., that there are about as many probes in deletions as there are probes in amplifications). In tumors where the balance between deletions and amplifications is heavily skewed, this assumption can lead to rescaled copy number estimates. For example, if a tumor carries a significant surplus of amplifications compared to deletions (e.g., in case of high hyperdiploidy), the CNs of deletions in the same genome may appear deeper than normal. As a theoretical example, the CN of a small two-copy deletion in an otherwise triploid genome (i.e., whole-genome duplication) will be about 2/3 (assuming 100% tumor cell fraction; and closer to 1.0 if lower fraction). While the latter would be an extreme case, our thresholds should still be appropriate, as for example 0.5 < 2/3 for TCGA.

In all, the thresholds we use to call homozygous deletions are motivated both by theoretical and distributional arguments. To call hemizygous deletions, which is not critical for identifying deletion limitation, we used a threshold of log_2_ ratio < −0.4.

### Simulation experiments

We performed simulation experiments to illustrate the distribution of regional chromosomal deletions under combined positive and negative selection. Here we computationally created copy number data for an artificial chromosome harboring a fictive deletion target and a set of neighboring essential and non-essential genes. To simulate the positive selection, we let the target gene be inactivated through various mutational mechanisms. The first copy of the target gene was allowed to be inactivated by regional deletion with breakpoints positioned randomly along the chromosome, whole-chromosome deletion, or point mutation. The second copy was allowed to be inactivated by the same mechanisms as well as reduplication of the mutant first chromosome combined by loss of the non-mutated chromosome. The mechanism of inactivation was selected randomly with fixed probabilities. To simulate negative selection, examples where both copies of a neighboring essential gene were hit by a deletion were rejected. Simulations were performed until 1000 acceptable examples had been generated.

### Calculation of deletion-limitation score

To calculate the deletion-limitation score (DLS) for a given gene, we first calculate the raw correlation between homozygous deletion frequency and genomic position across a fixed-size neighborhood upstream or downstream of the gene, and then selected the largest correlation observed in either direction as the final score. The upstream DLS for the gene becomes

1 $${\mathrm{DLS}}_{\mathrm{upstream}} = \frac{{\mathop {\sum }\nolimits_{i = 0}^N \,if\left( {x_{\mathrm{start}} - i} \right)}}{{\sqrt {\mathop {\sum }\nolimits_{i = 0}^N i^2\mathop {\sum }\nolimits_{i = 0}^N f\left( {x_{\mathrm{start}} - i} \right)^2} }}$$

and the downstream DLS for the same gene becomes

2 $${\mathrm{DLS}}_{\mathrm{downstream}} = \frac{{\mathop {\sum }\nolimits_{i = 0}^N if\left( {x_{\mathrm{end}} + i} \right)}}{{\sqrt {\mathop {\sum }\nolimits_{i = 0}^N i^2\,\mathop {\sum }\nolimits_{i = 0}^N f\left( {x_{\mathrm{end}} + i} \right)^2} }}$$

where *f* denotes the homozygous deletion frequency at a given genomic position, as calculated from the copy number data, *x*_start_ and *x*_end_ the p- and q-terminal positions of the gene. As our final score, we used the maximum of DLS_downstream_ and DLS_upstream_.

Although the DLS aims to quantify the degree of deletion limitation per gene basis, it is calculated by integrating information across a neighborhood with a certain size, which determines the degree of smoothing of information across nearby genes. A smaller neighborhood will capture deletion-limiting phenomena with less smoothing, while producing scores that are based on fewer deletion events. Conversely, a larger neighborhood will produce scores that are based on more deletion events, but with more smoothing across genes. We calculated DLS using neighborhood sizes of 0.5, 1, and 2 Mb. Because essential genes can be assumed never to be homozygously deleted (or at least at a low frequency; some hemizygous lesions may still be misclassified as homozygous), we calculated DLS for genes that were homozygously deleted in at most two cases in each copy number data set. Because of the smoothing, multiple genes can be identified as deletion-limiting at a specific locus. In such cases, we identified the candidate gene closest to the deletion target gene (deletion peak) as the limiting gene. To estimate the robustness of DLS scores, we used 200-fold bootstrapping.

### Statistics and reproducibility

The simulations and DLS score calculations were done using custom C++ programs.

### sgRNA oligo synthesis and pooled library cloning

We designed a custom sgRNA library targeting our locus of interest on chromosome 9p21 flanking *CDKN2A*. sgRNA sequences for all 68 genes spanning from 16.5 (BNC2) to 34 Mb (UBAP2) on chromosome 9p were obtained from the human GeCKO v2.0. library^40^. Our sgRNA pool consisted of 398 sgRNAs sequences (4–6 sgRNAs per gene, Supplementary Table 2) synthesized as standard desalted DNA oligos (Integrated DNA Technologies), phosphorylated with T4 PNK (Thermo Fisher Scientific), pair-wise annealed and mixed in an equimolar manner. The lentiCRISPRv2 plasmid (#52961, Addgene) was digested with the FastDigest Esp3I restriction enzyme (Thermo Fisher Scientific) and gel purified (Macherey-Nagel). Plasmid vector and sgRNA pool were mixed at a 1:8 ratio and ligated using the Rapid DNA Ligation kit (Thermo Fisher Scientific). Unligated plasmid was digested using Plasmid Safe Exonuclease (EpiCentre), and subsequently the ligated product was purified using the DNA Clean & Concentrator™-5 (Zymo Research). MegaX DH10B electrocompetent cells (Thermo Fisher Scientific) were transformed with 2 µl ligated product by electroporation using a GenePulser II (BioRad) (settings: 2.0 kV, 200 Ω and 25 µF) in duplicates, and cells were resuspended to 1 ml S.O.C. recovery medium (Thermo Fisher Scientific) and incubated for 1 h at 37 °C (225 rpm). Duplicates were subsequently pooled and plated onto 10 cm^2^ agar plates with ampicillin selection (50 µg/ml), which yielded 175X library coverage. After 20 h of incubation at 32 °C, colonies were scraped off and combined, and plasmid DNA was extracted using Endotoxin-Free Plasmid Maxiprep (Qiagen). The baseline distribution of sgRNAs in the plasmid pool was determined by single-end next-generation sequencing, and 99.5% (396/398) of sgRNA sequences were successfully represented in the final library.

### Lentivirus production

Lentiviral production was performed as described before^16^ with modifications. Briefly, HEK293T cells were seeded at ~40% confluence in T175 flasks one day before transfection in DMEM medium (Invitrogen) supplemented with 10% Gibco Fetal Bovine Serum (FBS, Thermo Fisher Scientific) and 1X penicillin-streptomycin mix. The media was replaced by 13 ml OptiMEM (Invitrogen) 1 h prior to transfection. For each transfection, 20 µg lentiCRISPR plasmid library were co-transfected with packaging plasmids (10 µg pMD2.G (#12259), 15 µg psPAX2 (#12260, Addgene)), 200 µl Plus reagent and 4 µl OptiMEM (Invitrogen). After 5 min of incubation at room temperature, a Lipofectamine mixture (100 µl Lipofectamine 2000 diluted in 4 ml optiMEM (Invitrogen)) was added to the plasmid mixture and incubated for 20 min at room temperature, before being added drop wise to the HEK293T cells. Cells were incubated at 37 °C, 5% CO_2_ and the medium was replaced with 30 ml fresh DMEM (Invitrogen) supplemented with 10% FBS (Thermo Fisher Scientific), 1% BSA (Sigma Aldrich) and 1× penicillin/streptomycin after 6 and 24 h post-transfection. Viral supernatant was harvested at 48, 72, and 96 h post transfection. The supernatant was centrifuged at 2000 rpm at 4 °C for 10 min, filtered through a 0.45 µm low protein binding membrane (Merck) and stored at 4 °C until all harvests had been completed. Finally, virus supernatants were precipitated using PEG virus precipitation kit (AH Diagnostics), aliquoted and stored at −80 °C.

### Cell culture and cell transduction using the sgRNA library

Pooled lentiviral libraries were transduced into relevant ALL cell lines identified via the Cancer Cell Line Encyclopedia (CCLE). RCH-ACV (ACC 548, DSMZ) is a B-ALL cell line showing no copy number variation on chromosome 9, while Jurkat (ACC 282, DSMZ) is a T-ALL cell line with a 2.3 Mb homozygous regional deletion containing CDKN2A and a truncating *TP53* mutation. Both cell lines were cultured in RPMI-1640 supplemented with 10% FCS (RPMI-10) and maintained at a density of 0.5 million cells/ml.

Cells were transduced in triplicates at an average multiplicity of infection (MOI) of ~0.3 (0.23–0.35 and 0.25–0.41, respectively) to minimize the risk of multiple sgRNA integration in single cells. One million cells per well were seeded in 1 ml RPMI-10 supplemented with 8 µg/ml polybrene (Sigma Aldrich) and transduced in multiple wells of a 12-well plate along with a no-transduction control. The plate was centrifuged at 2300 rpm for 30 min at 37 °C and incubated overnight. After 24 h RCH-ACV cells were supplemented with 1 ml RPMI-10, while Jurkat cells were pelleted and resuspended in 2 ml RPMI-10. After 48 h (day 0), cells from all wells were combined and medium was replaced with fresh RPMI-10 supplemented with puromycin for 72 h (0.75 µg/ml for RCH-ACV and 1 µg/ml for Jurkat). A fraction of the cell suspension (2.5–5 × 10^6^ cells) was sampled every 48 to 72 h (days 0, 3, 5, 7, 10, 12, and 14) for DNA extraction, while the remaining cells were maintained in RPMI-10 supplemented with puromycin.

### Pooled sgRNA screening and data analysis

Genomic DNA was extracted for all timepoints using the QIAamp Blood DNA Mini kit (Qiagen). A step of PCR was performed to amplify lentiCRISPR sgRNAs from genomic DNA and attach Illumina adaptors and indexes to the samples (Herculase II Fusion DNA polymerase (Agilent), NEBNext® Ultra™ II Q5® Master Mix (New England Biolabs)). Forward primers include a variable length sequence to increase library complexity, while reverse primers include a 10-bp index sequence to facilitate multiplexing. Primer sequences used are shown in Table 2. The amount of input genomic DNA (gDNA) per reaction required to achieve a 500-fold representation of each sgRNA was 1.31 µg (500× coverage * 398 sgRNAs * 6.6 pg [average DNA weight per cell]), after compensating for cell viability. PCR amplification was carried out with 22 cycles and PCR products were gel extracted (Nucleospin Gel and PCR Cleanup, Macherey-Nagel), quantified (Qubit™ dsDNA HS Assay Kit, Thermofischer Scientific), combined in an equimolar manner and sequenced on a HiSeq 2500 (Illumina).

**Table 2:** sgRNA amplification primers

sgRNA primer	Sequence
Forward	5′-AATGATACGGCGACCACCGAGATCTACACTCTTTCCCTACACGACGCTCTTCCGATCT - (1–9 bp variable length sequence) - tcttgtggaaaggacgaaacaccg -3′
Reverse	5′-CAAGCAGAAGACGGCATACGAGAT – 10 nt Index – GTGACTGGAGTTCAGACGTGTGCTCTTCCGATCTtgtgggcgatgtgcgctctg -3′

Raw FASTQ files were demultiplexed and read quality was checked with FastQC^48^. After stringent mapping to the reference sgRNA library using BWA (http://bio-bwa.sourceforge.net/), the number of uniquely aligned reads per sgRNA was calculated and normalized. The representation of each sgRNA was assessed by deep sequencing of integrated sgRNA sequences at days 3, 5, 7, 10, 12, and 14 post-puromycin selection after normalizing the read count for each sgRNA to the total read count. To infer either depletion or enrichment of specific sgRNA species, the read counts for each sgRNA at each timepoint were normalized to the read counts at day 3 (after 72 h of puromycin selection).

### CRISPR-Induced Insertion/Deletion Detection

To detect CRISPR-induced indel mutations, we designed PCR primers flanking the sgRNA target sites for a selection of 18 genes. We amplified 150 to 270 bp amplicons centered on the sgRNA recognition site in multiplex PCR reactions of 6–7 targets (Supplementary Table 5). Groups of amplicons were determined using the MultiPLX 2.1 tool^49^ and 100 ng of genomic DNA was amplified for 25 cycles with the KAPA2G Fast Multiplex PCR Kit (Sigma Aldrich). Genomic DNA from non-transduced cells was used as a control to determine PCR or sequencing errors, while gDNA from transduced cells from day 3 and day 7 was used to quantify the abundance of CRISPR-induced indels. All reactions were performed in triplicates.

PCR products were quantified and pooled in an equimolar manner, followed by purification. Library preparation was performed with the NEBNext® Ultra™ II DNA Library Prep Kit for Illumina (New England Biolabs) following the manufacturer’s protocol but using custom adaptors. Briefly, 200 ng of PCR product was end repaired and ligated with custom barcoded Illumina adaptors, followed by purification and 3 PCR cycles to enrich the adaptor-ligated DNA. Barcoded libraries were pooled equimolarly and sequenced on a HiSeq 2500 (Illumina) using paired-end 125 bp sequencing.

Reads were aligned to the UCSC hg19 reference genome, collected from the BSGenome package^50^ and the CrispRVariants Bioconductor package^51^ was used to perform variant counting and visualization. We searched for indels/SNVs within a range of 35 bases flanking each sgRNA sequence (27 bases upstream and 8 bases downstream the PAM site). We then calculated variant counts and plotted each sgRNA with CrispRVariants built-in commands. To remove common PCR-induced mutations from the analysis, observed sequences that occurred in the corresponding control samples (non-transduced) were discarded.

### Reporting summary

Further information on research design is available in the Nature Research Reporting Summary linked to this article.

## Supplementary information

Supplementary Information

Reporting Summary

## Data Availability

Segmented copy number data for 7,268 primary tumor samples belonging to 24 cancer types from The Cancer Genome Atlas (TCGA) (http://cancergenome.nih.gov/), 2476 samples belonging to 39 cancer types from the Tumorscape compendium (http://www.broadinstitute.org/tumorscape) and 1043 samples representing human cancer cell lines from the Cancer Cell Line Encyclopedia (https://portals.broadinstitute.org/ccle). DepMap gene dependency scores were obtained from https://depmap.org/portal/download/all/ (DepMap Public 19Q1 release). The remaining data are contained within the paper and Supplementary Files or are available from the authors upon request.

## Code availability

Computer code is available on GitHub (https://github.com/LudvigEk/tsimchr) and request from the corresponding author.
