# Supplementary material for: Essential genes shape cancer genomes through linear limitation of homozygous deletions
Source: Commun Biol. 2019 Jul 19;2:262. doi: 10.1038/s42003-019-0517-0 (PMC6642121; doi:10.1038/s42003-019-0517-0)
Supplement: Supplementary file 1 — Supplementary Information [file 42003_2019_517_MOESM1_ESM.pdf]

Supplementary Figure 1

Additional simulation plots. These additional simulation plots were carried out in the same way as in Fig. 1, but with different parameter settings: **(a)** 1% chance of regional deletions, 2% chance for point mutations and 1% chance of reduplication, **(b)** 5% chance of regional deletions, 5% chance for point mutations and 20% chance of reduplication and **(c)** 50% chance of regional deletions, 1% chance for point mutations and 1% chance of reduplication. These additional simulation illustrate that, although changing the parameter settings may change the frequency of hemizygous and homozygous deletion frequencies, the linear deletion limitation pattern at the EGs closest to the deletion target persists, independently of parameter settings.

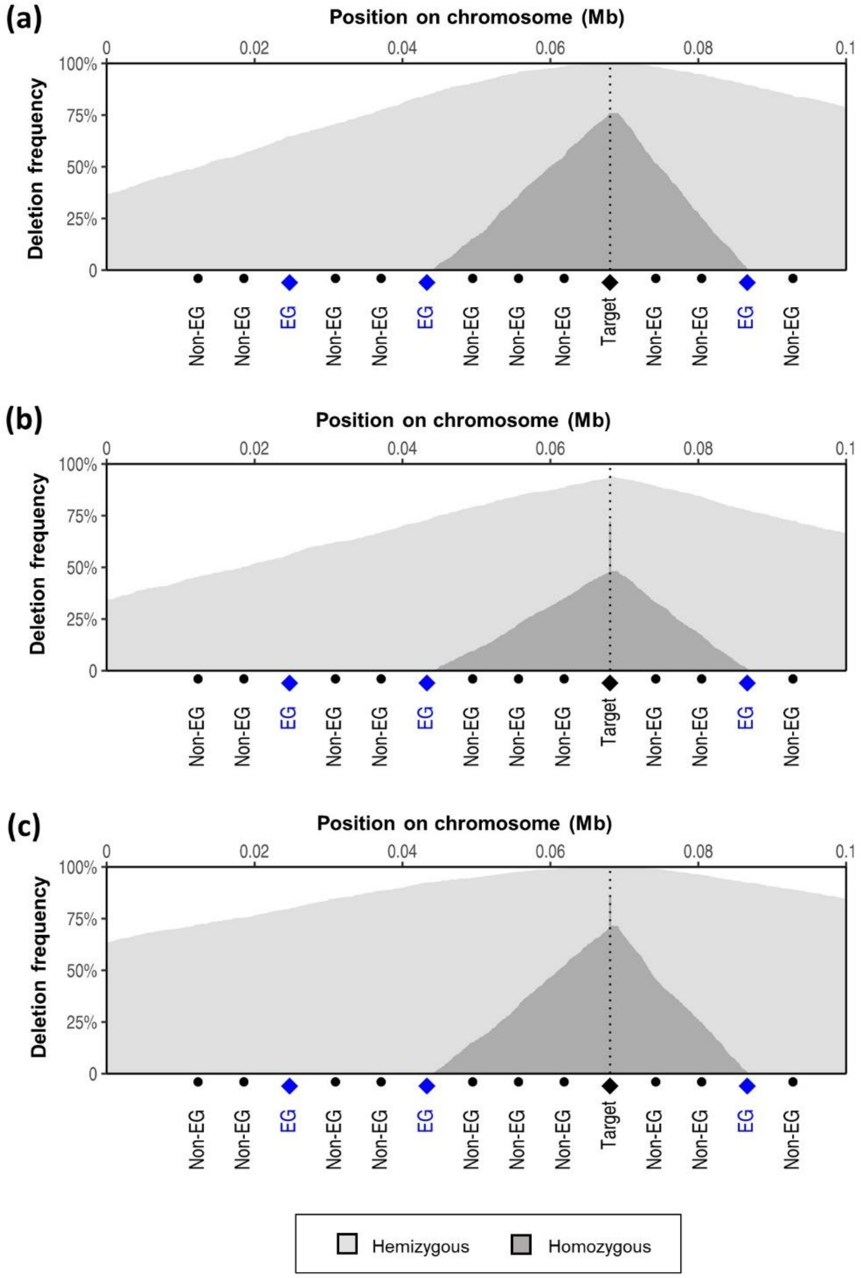

Supplementary Figure 2

Distributions of observed copy numbers *vs* size for deletions in TCGA **(a)**, CCLE **(b)**, and Tumorscape **(c)**. The dashed lines indicate the positions of the thresholds used to call homozygous deletions. These thresholds were positioned left of where the distributions of very large lesions thin out. As discussed in detail in Methods, the motivation for this is that very large lesions (in the order of 50-100 million base pairs; corresponding to whole chromosomes or big parts of chromosomes) are most often hemizygous since the probability that they include at least one essential gene is close to 1.

(a)

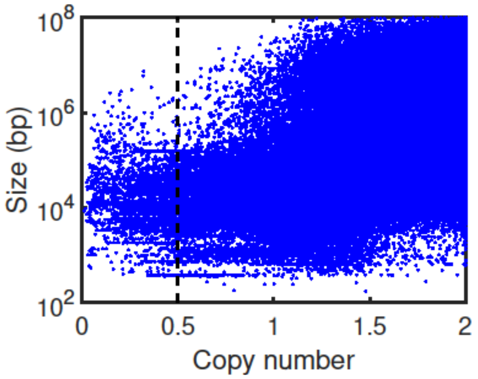

(b)

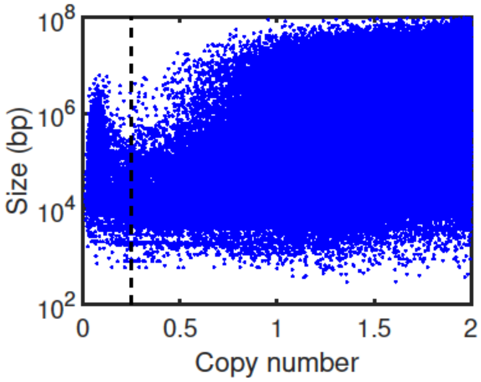

(c)

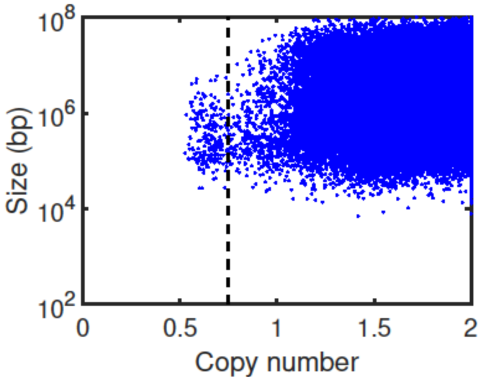

Supplementary Figure 3

Estimated tumor cell fractions for TCGA **(a)**, CCLE **(b)**, and Tumorscape **(c)**. The median estimated fractions were 89%, 99%, and 58%, respectively.

(a)

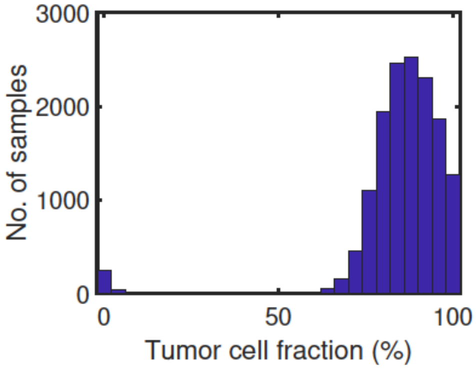

(b)

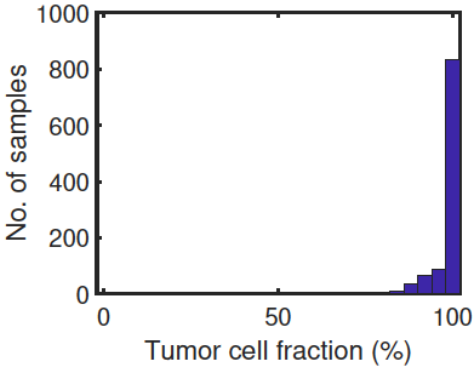

(c)

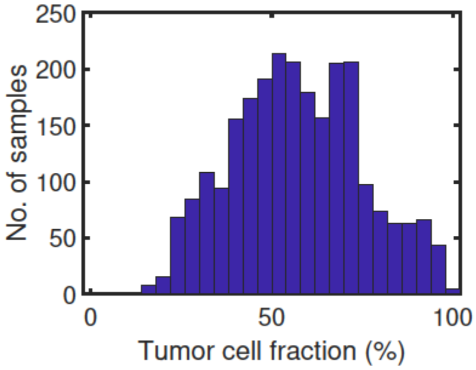

Supplementary Figure 4

Deletion frequencies of target genes. Heatmap showing the deletion frequencies of the likely target genes in the regions harboring the identified deletion-limiting genes. Data from TCGA[21] and Tumorscape[23]. BLCA: Bladder Carcinoma, BRCA: Breast Carcinoma, COAD: Colon Adenocarcinoma, DLBC: Diffuse Large B-cell Lymphoma, GBM: glioblastoma, HNSC: Head and Neck Squamous Cell Carcinoma, KICH: Kidney Chromophobe, KIRC: Kidney Renal Clear Cell Carcinoma, KIRP: Kidney Renal Papillary Cell Carcinoma, LAML: Acute Myeloid Leukemia, LGG: Brain Lower Grade Glioma, LIHC: Liver Hepatocellular Carcinoma, LUAD: Lung Adenocarcinoma, LUSC: Lung Squamous Cell Carcinoma, OV: Ovarian Serous Cystadenocarcinoma, PAAD: Pancreatic Adenocarcinoma, PRAD: Prostate Adenocarcinoma, READ: Rectum Adenocarcinoma, SARC: Sarcoma, SKCM: Skin Cutaneous Melanoma, STAD: Stomach Adenocarcinoma, THCA: Thyroid Carcinoma, UCEC: Uterine Corpus Endometrial Carcinoma, ALL: Acute Lymphoblastic Leukemia, GI: Gastrointestinal (COAD, esophageal, gastric, gastrointestinal stromal tumor), KI: Kidney (KICH, KIRC, KIRP, oncocytoma), LI: Liver (LIHC), LU: Lung (LUAD, LUSC, bronchioloalveolar carcinoma, large cell carcinoma, mesothelioma), MDS: Myelodysplastic syndrome, MPD: Myeloproliferative Disorder, CNS: Central Nervous System (glioma, medulloblastoma, meningioma, neuroblastoma, rhabdoid, schwannoma), OV: Ovarian, PR: Prostate cancer.

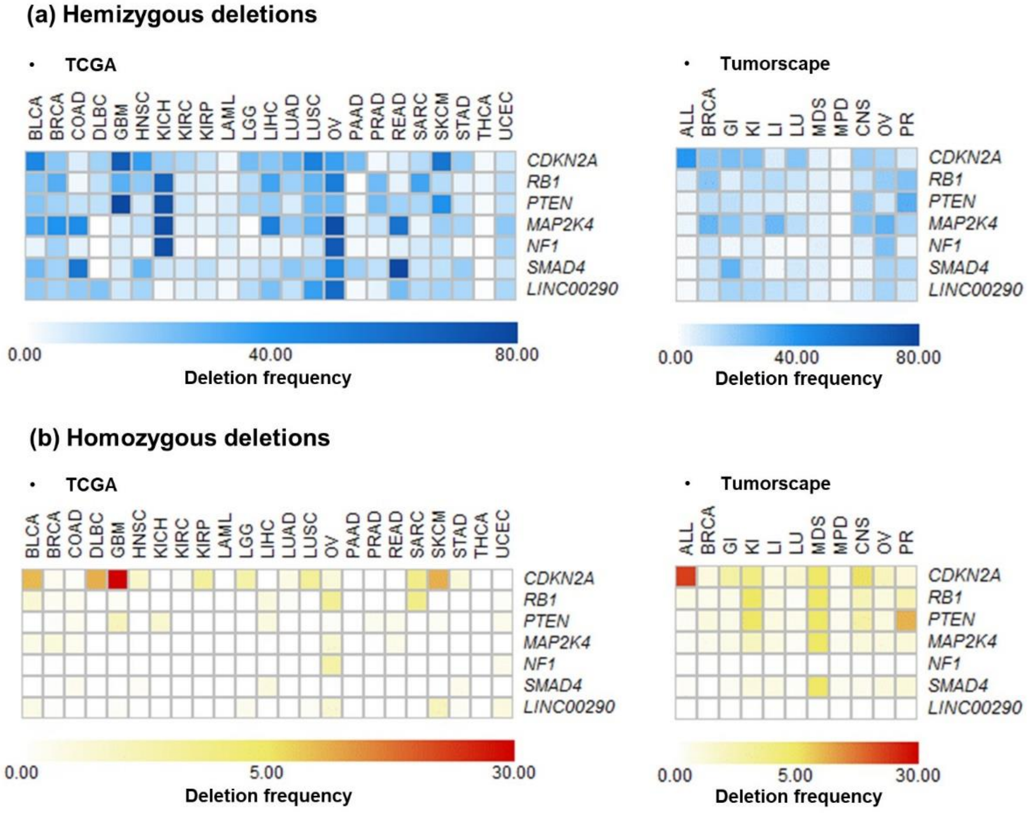

Supplementary Figure 5

Patterns of hemizygous and homozygous deletion across copy number profiles of 1,043 cancer cell lines from the Cancer Cell Line Encyclopedia (CCLE) in the seven genomic regions showing deletion-limitation in TCGA: *CDKN2A* **(a)**, *RB1* **(b)**, *MAP2K4* **(c)**, *NF1* **(d)**, *SMAD4* **(e)**, *LINC00290* **(f)** and *PTEN* **(g)**.

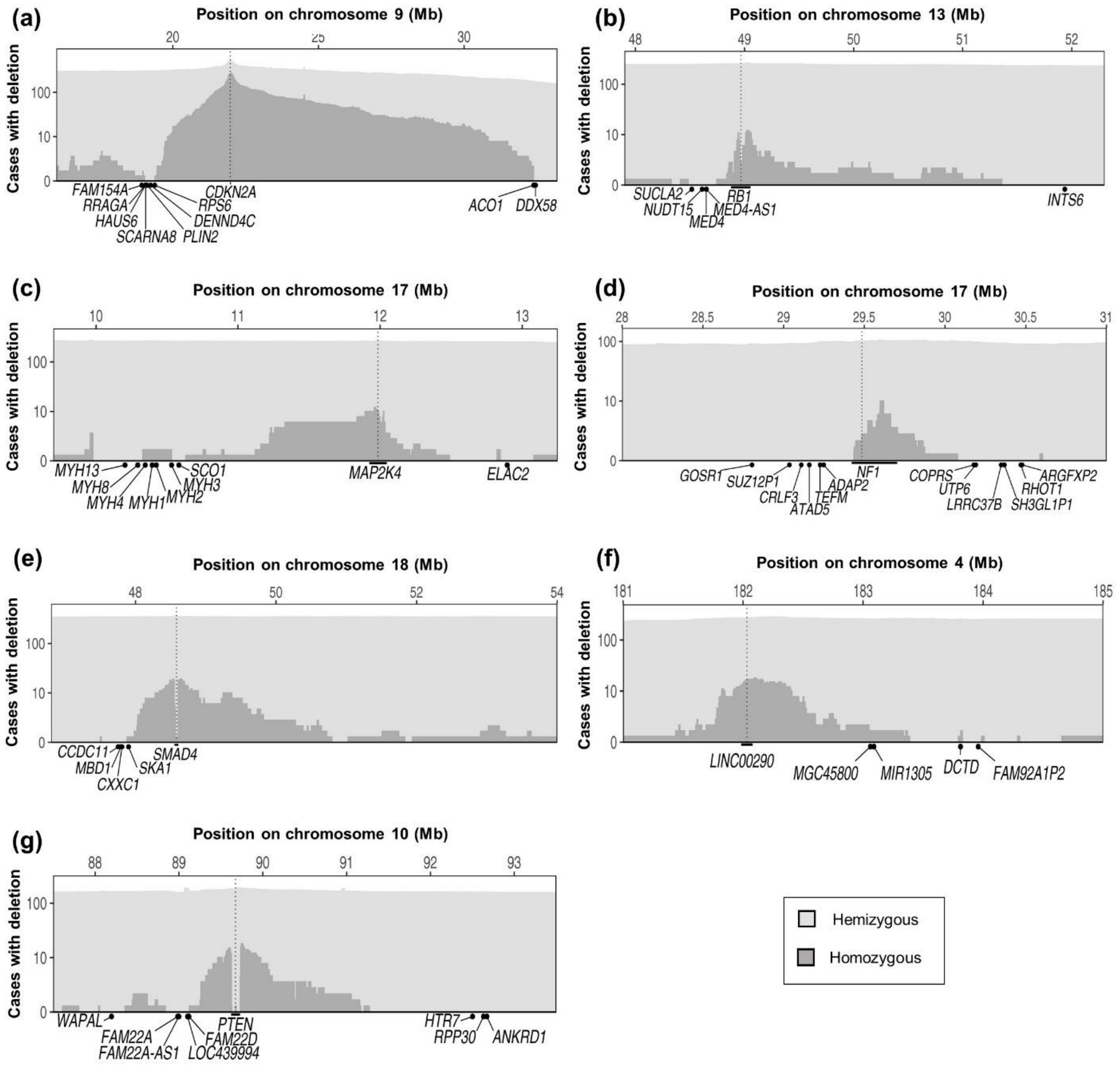

Supplementary Figure 6

Patterns of hemizygous and homozygous deletion across copy number profiles of 2,476 samples from Tumorscape for the seven regions where we identified deletion limitation in TCGA and CCLE. In Tumorscape, we observed deletion patterns similar to those observed in TCGA and CCLE at *CDKN2A* **(a)**, *MAP2K4* **(c)** and *PTEN* **(g)**, whereas the *RB1* region **(b)** showed slightly different patterns on the *p*-terminal side and for the remaining loci *NF1* **(d)**, *SMAD4* **(e)** and *LINC00290* **(f)** there were not sufficient cases with homozygous deletions.

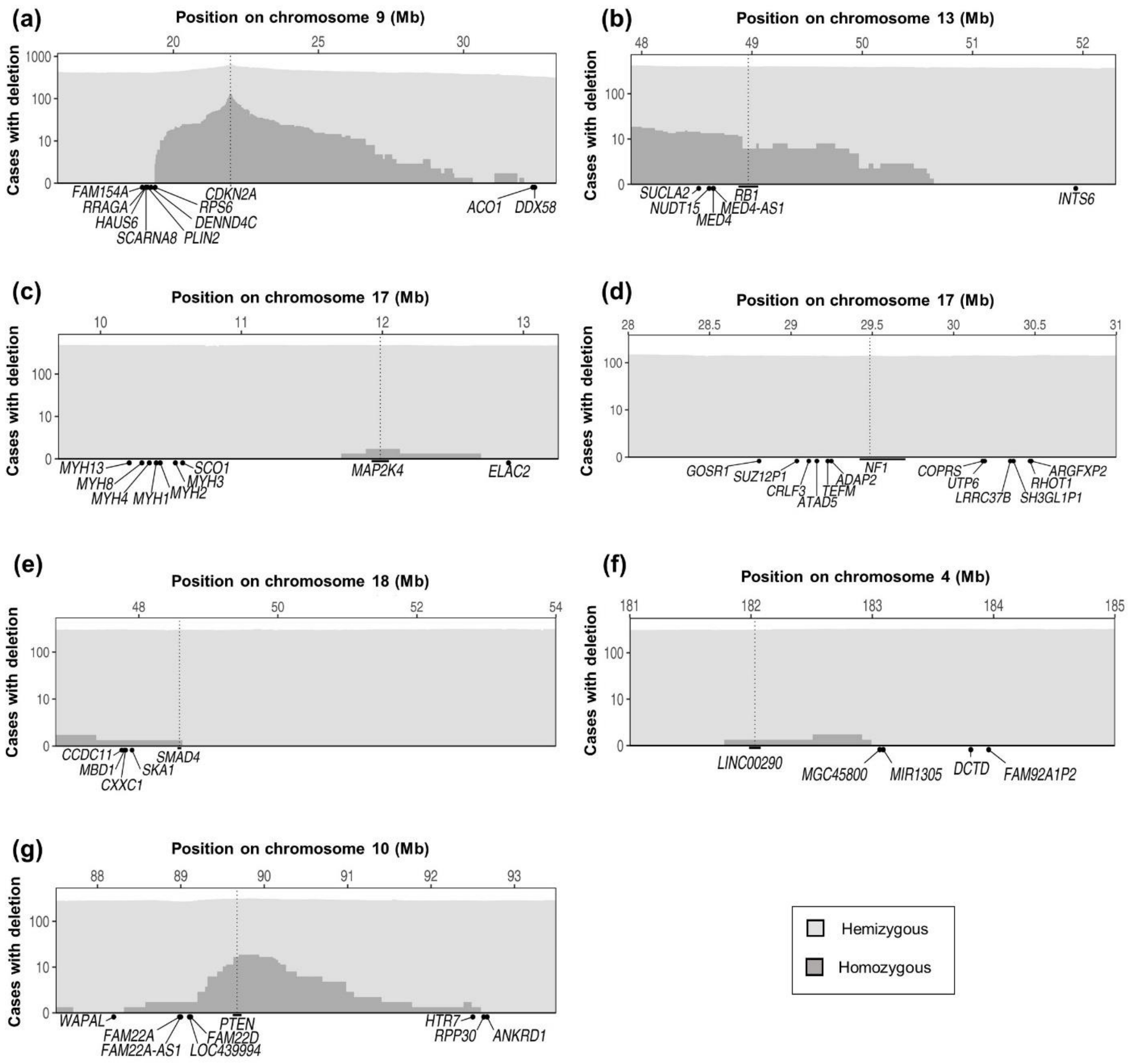

Supplementary Figure 7

Mapping of EGs in the *CDKN2A* region using CRISPR/Cas9 in Jurkat cells. The strongest DLS scores were found in the *CDKN2A* region on 9p21. For further validation of essentiality, and to clarify which are essential on the q-terminal side, we carried out a focused CRISPR/Cas9 screen of 68 genes in the region in Jurkat and RCH-ACV cells. **(a)** Overview of hemizygous and homozygous deletion frequency across the *CDKN2A* region (TCGA dataset). **(b)**, **(c)** Detailed overview of the p- and q-terminal deletion limit points. Black dots at the lower part of the figure represent sgRNA sequences showing <25% depletion by day 14. Blue diamonds illustrate sgRNAs showing >25% depletion. For the p-terminal conserved region, we saw depletion of sgRNAs towards *HAUS6* and *RPS6*. For the q-terminal conserved region, we saw depletion of sgRNAs towards *DNAJA1*, *SMU1* and *NOL6*.

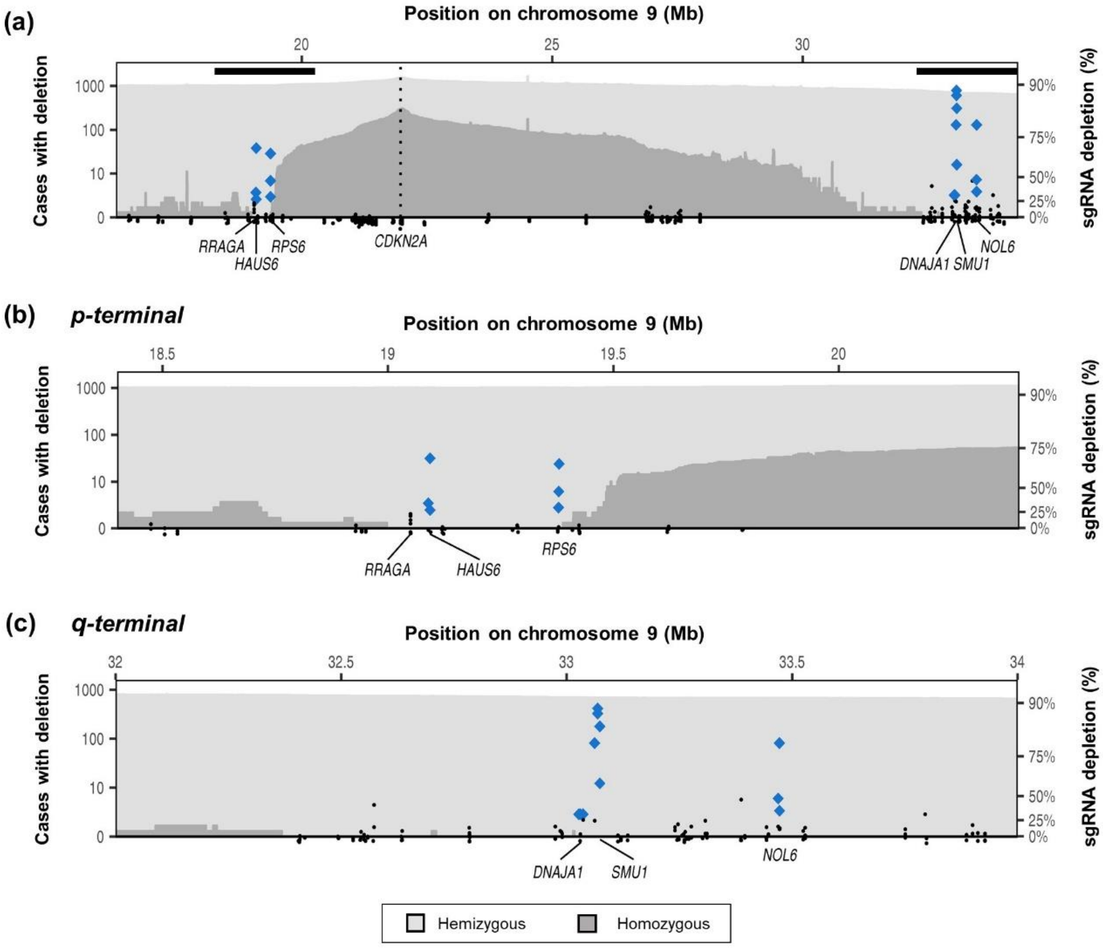

Supplementary Figure 8

Mapping of EGs in the *CDKN2A* region using CRISPR/Cas9 in RCH-ACV cells. (a) Overview of hemizygous and homozygous deletion frequency across the *CDKN2A* region (TCGA dataset). **(b)**,**(c)** Detailed overview of the p- and q-terminal deletion limit points. Black dots at the lower part of the figure represent sgRNA sequences with a depletion lower than 25% by day 14. Blue diamonds illustrate sgRNAs showing >25% depletion. In the p-terminal conserved region, we observed depletion of sgRNAs towards *RRAGA*, *HAUS6* and *RPS6*. In the q-terminal conserved region, we observed depletion of sgRNAs towards *SMU1* and *NOL6*.

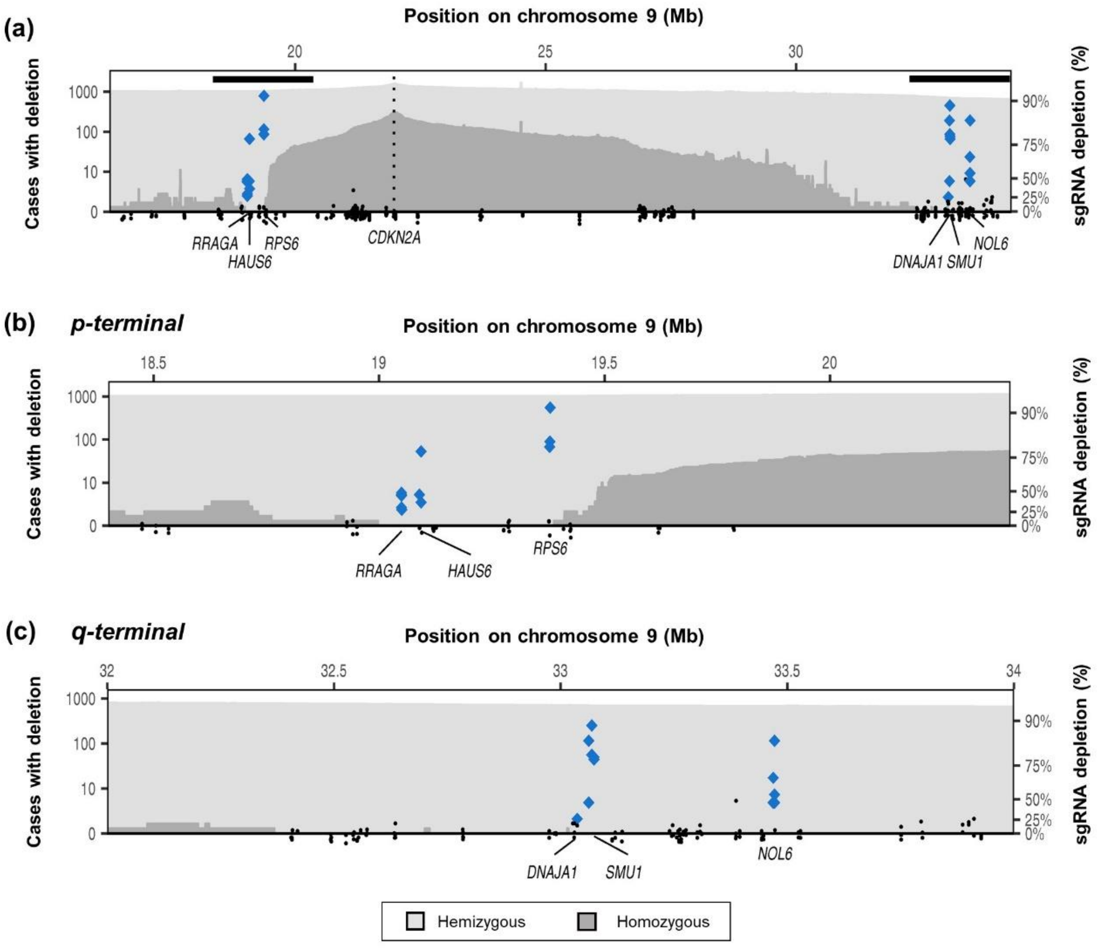

Supplementary Figure 9

Associations between gene expression and DNA copy number. Correlation plots between copy number and mRNA expression for the identified deletion-limiting genes. As shown, hemizygous deletion of these genes reduces expression in most cases.

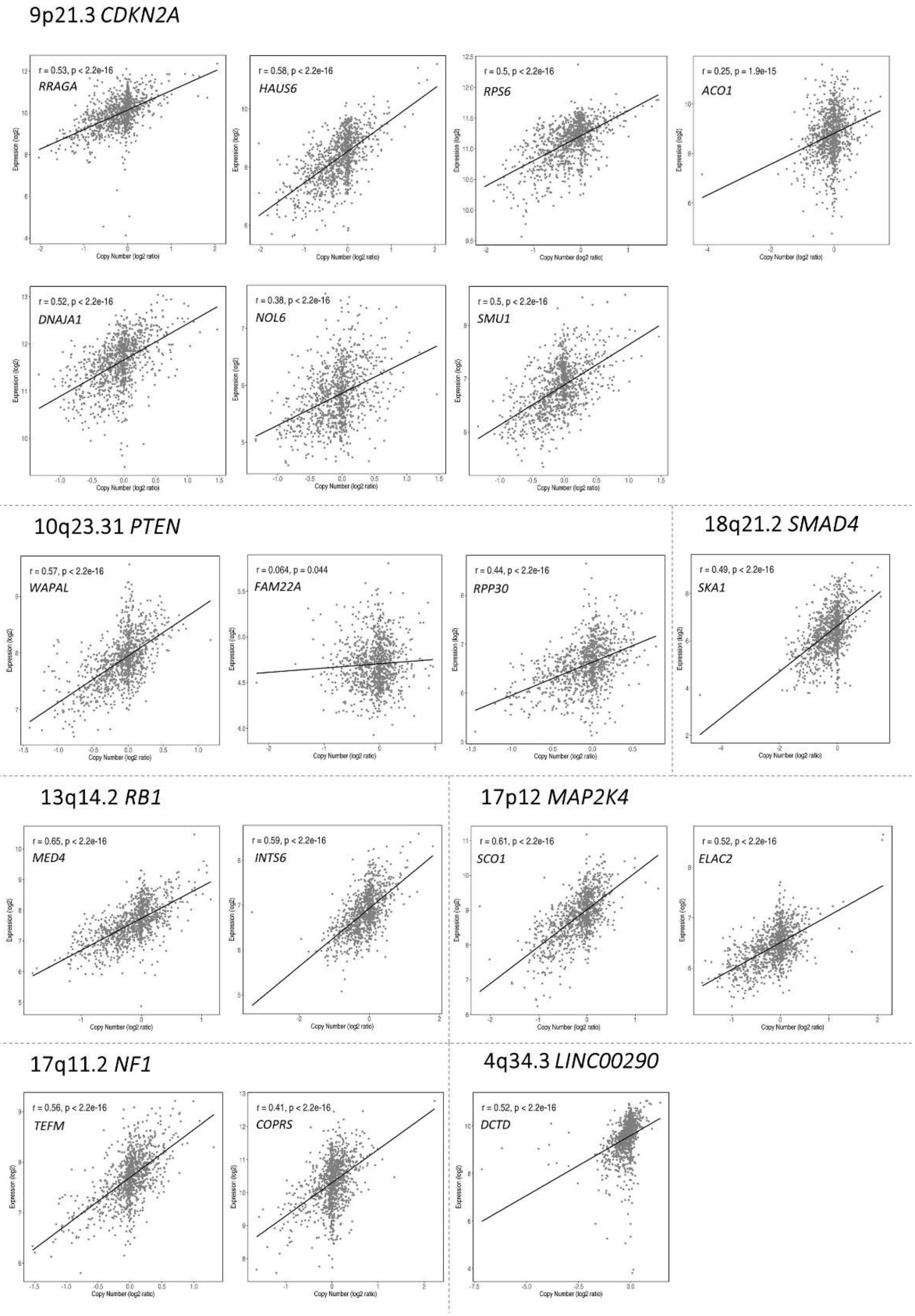

Supplementary Table 1

DLS scores in the CCLE and Tumorscape datasets. The candidate deletion-limiting genes identified in the TCGA dataset show a DLS>0.8 (*r*) within a 0.5-, 1- and/or 2-Mb neighborhood. Positive and negative *r*-values indicate candidate genes at the *p*- and q-terminal, respectively. Using 200-fold bootstrapping, we estimated 90% confidence intervals (90%CI).

				0.5Mb						1Mb						2Mb					
				CCLE			Tumorscape			CCLE			Tumorscape			CCLE			Tumorscape		
**GeneSymbol**	**Chr**	**Start**	**End**	**r**	**90%CI**	***N***	**r**	**90%CI**	***N***	**r**	**90%CI**	***N***	**r**	**90%CI**	***N***	**r**	**90%CI**	***N***	**r**	**90%CI**	***N***
**4q34.3 *LINC00290***																					
*LOC90768**	4	183060158	183065668							-0,979	[-0.984, -0.921]	17	-0,756	[-0.918, 0]	2						
*MIR1305**	4	183090445	183090531							-0,976	[-0.983, -0.912]	17	-0,773	[-0.925, 0]	2						
DCTD	4	183811243	183838630													-0,927	[-0.951, -0.864]	17	-0,835	[-0.931, 0.685]	2
*FAM92A1P2**	4	183958817	183961272													-0,835	[-0.925, -0.734]	15	-0,832	[-0.911, 0.635]	2
**9p21.3 *CDKN2A***																					
FAM154A	9	18927890	19033256							0,782	[0.481, 0.910]	12	0,974	[0.943, 0.977]	17	0,952	[0.902, 0.976]	49	0,990	[0.974, 0.993]	28
RRAGA	9	19049371	19051021							0,793	[0.514, 0.912]	12	0,978	[0.949, 0.98]	18	0,953	[0.899, 0.977]	51	0,990	[0.973, 0.994]	28
HAUS6	9	19053134	19102940							0,829	[0.604, 0.933]	12	0,987	[0.963, 0.987]	20	0,958	[0.907, 0.980]	51	0,991	[0.973, 0.994]	31
*SCARNA8**	9	19063653	19063784							0,801	[0.536, 0.917]	12	0,980	[0.953, 0.982]	18						
PLIN2	9	19115758	19127604							0,871	[0.737, 0.951]	13	0,990	[0.97, 0.989]	20	0,961	[0.911, 0.981]	54	0,990	[0.973, 0.994]	31
DENND4C	9	19230762	19374137	0,978	[0.845, 0.986]	8	0,978	[0.944, 0.989]	16	0,965	[0.909, 0.986]	21	0,985	[0.959, 0.993]	23	0,963	[0.930, 0.980]	83	0,983	[0.964, 0.99]	46
RPS6	9	19376253	19380235	0,980	[0.852, 0.986]	8	0,977	[0.943, 0.988]	16	0,977	[0.926, 0.988]	22	0,984	[0.958, 0.993]	23	0,959	[0.919, 0.977]	83	0,983	[0.964, 0.99]	47
ACO1	9	32384600	32450832													-0,973	[-0.986, -0.926]	13	-0,495	[-0.495, -0.0831]	2
DDX58	9	32455299	32526322													-0,977	[-0.987, -0.934]	13	-0,534	[-0.534, -0.0985]	2
**10q23.31 *PTEN***																					
WAPAL	10	88195012	88281541													0,831	[0.727, 0.864]	17	0,921	[-0.22, 0.972]	16
FAM22A	10	88985204	88994733	0,905	[0.660, 0.956]	6	0,980	[0.87, 0.984]	7	0,880	[0.822, 0.894]	17	0,989	[0.96, 0.99]	17						
*FAM22A-AS1**	10	88998423	89102315	0,964	[0.700, 0.982]	11	0,995	[0.946, 0.993]	9	0,859	[0.775, 0.892]	17	0,981	[0.952, 0.987]	17						

LOC439994	10	89102167	89103331	0,964	[0.702, 0.982]	11	0,991	[0.939, 0.99]	8	0,858	[0.774, 0.892]	17	0,980	[0.952, 0.986]	17						
FAM22D	10	89117476	89130452	0,953	[0.729, 0.967]	11	0,994	[0.951, 0.992]	8	0,844	[0.751, 0.885]	17	0,975	[0.943, 0.983]	17						
HTR7	10	92500575	92617671													-0,842	[-0.853, -0.526]	5	-0,969	[-0.98, -0.845]	7
RPP30	10	92631708	92668312													-0,789	[-0.803, -0.366]	4	-0,960	[-0.975, -0.79]	7
ANKRD1	10	92671856	92681032													-0,769	[-0.784, -0.359]	4	-0,958	[-0.976, -0.773]	7
**13q14.2 *RB1***																					
SUCLA2	13	48516790	48575462	0,859	[0.734, 0.925]	10	-0,917	[-0.965, -0.774]	9												
NUDT15	13	48611702	48621282	0,895	[0.746, 0.950]	10	-0,891	[-0.961, -0.688]	9												
MED4	13	48649863	48669277	0,896	[0.696, 0.954]	10	-0,886	[-0.965, -0.672]	7												
*MED4-AS1**	13	48651272	48654129	0,898	[0.710, 0.956]	10	-0,886	[-0.964, -0.673]	7												
**17p12 *MAP2K4***																					
MYH13	17	10204182	10276322													0,891	[0.780, 0.940]	11	0,747	[0, 0.79]	2
MYH8	17	10293641	10325267													0,873	[0.751, 0.933]	11	0,764	[0, 0.812]	2
MYH4	17	10346607	10372876													0,857	[0.726, 0.925]	11	0,779	[0, 0.831]	2
MYH1	17	10395626	10421859													0,835	[0.698, 0.908]	11	0,792	[0, 0.849]	2
MYH2	17	10424464	10452940													0,820	[0.681, 0.899]	11	0,800	[0, 0.859]	2
MYH3	17	10531842	10560626													0,755	[0.623, 0.834]	11	0,823	[0, 0.889]	2
SCO1	17	10583648	10600885													0,727	[0.600, 0.805]	11	0,831	[0, 0.898]	2
ELAC2	17	12894928	12921381							-0,869	[-0.939, -0.675]	11	-0,969	[-0.969, 0]	2						
**17q11.2 *NF1***																					
GOSR1	17	28804425	28853832							0,827	[0.713, 0.856]	9	0	[0, 0]	0						
SUZ12P1	17	29036625	29097068	0,812	[0.663, 0.821]	8	0	[0, 0]	0												
CRLF3	17	29109701	29151778	0,882	[0.76, 0.892]	9	0	[0, 0]	0												
ATAD5	17	29159022	29222295	0,922	[0.82, 0.937]	9	0	[0, 0]	0												
TEFM	17	29226000	29233286	0,921	[0.813, 0.939]	9	0	[0, 0]	0												
ADAP2	17	29248753	29286211	0,910	[0.778, 0.952]	9	0	[0, 0]	0												
COPRS	17	30178883	30186326	-0,959	[-0.979, -0.81]	5	0	[0, 0]	0												
LRRC37B	17	30348154	30380519							-0,732	[-0.803, 0.595]	8	0	[0, 0]	0						

*SH3GL1P1**	17	30367354	30369851							-0,752	[-0.827, -0.490]	8	0	[0, 0]	0						
RHOT1	17	30469472	30552746							-0,827	[-0.920, -0.574]	8	0	[0, 0]	0						
*ARGFXP2**	17	30477386	30478590							-0,826	[-0.922, -0.599]	8	0	[0, 0]	0						
**18q21.2 *SMAD4***																					
CCDC11	18	47753562	47792865							0,935	[0.902, 0.940]	18	-0,950	[-0.962, -0.399]	2						
MBD1	18	47793251	47808144							0,934	[0.901, 0.940]	18	-0,954	[-0.965, -0.221]	2						
CXXC1	18	47808712	47814692							0,933	[0.900, 0.940]	18	-0,955	[-0.966, -0.0574]	2						
SKA1	18	47901391	47920538							0,903	[0.851, 0.925]	18	-0,959	[-0.968, 0]	2						

* *non protein coding gene*

Supplementary Table 2

Gene dependency scores for the identified deletion-limiting genes. This table show data from genome-wide CRISPR loss-of-function screens from the Cancer Dependency Map[20,38] (DepMap AVANA 19Q1; 558 cell lines) and pooled shRNA knockdown screens[2,5,47] (shARP[5] and Achilles[47]) for the identified genes. Negative AVANA scores indicate depletion by sgRNA targeting the gene during culture. Low mean Achilles and shARP scores in copy number loss (CNL) compared to copy number-neutral (CN) cell lines indicates copy number-dependent sensitivity to further suppression of the activity of a gene by shRNA.

				DepMap	Achilles		shARP	
**GeneSymbol**	**Chr**	**Start**	**End**	**Dependency score**	**CNL mean dependence score**	**CN mean dependence score**	**CNL mean dependence score**	**CN mean dependence score**
**4q34.3 *LINC00290***				-	-	-	-	-
*LOC90768 **	4	183060158	183065668	-	-	-	-	-
*MIR1305 **	4	183090445	183090531	-	-	-	-	-
DCTD	4	183811243	183838630	0,1280	-0,076	-0,024	0,262	0,243
*FAM92A1P2 **	4	183958817	183961272	-	-	-	-	-
**9p21.3 *CDKN2A***								
FAM154A	9	18927890	19033256	-0,162	-	-	-	-
RRAGA	9	19049371	19051021	-0,376	-0,021	0,043	-	-
HAUS6	9	19053134	19102940	**-0,853**	-	-	-0,839	0,623
*SCARNA8 **	9	19063653	19063784	-	-	-	-	-
PLIN2	9	19115758	19127604	-0,136	-	-	-	-
DENND4C	9	19230762	19374137	0,002	-	-	-	-
RPS6	9	19376253	19380235	**-1,259**	-0,071	-0,165	**-0,769**	**1,084**
ACO1	9	32384600	32450832	0,088	-0,243	-0,129	-0,255	0,029
DDX58	9	32455299	32526322	-0,019	-	-	-	-
DNAJA1	9	33025211	33039907	-0,408	-	-	-0,592	-0,017
SMU1	9	33041764	33076716	**-1,411**	**-0,721**	0,164	**-2,006**	0,796
NOL6	9	33461353	33473943	**-0,930**	-	-	-0,049	-0,143
**10q23.31 *PTEN***								
WAPAL	10	88195012	88281541	-0,221	-	-	-	-
FAM22A	10	88985204	88994733	-0,172	-	-	-	-
*FAM22A-AS1 **	10	88998423	89102315	-	-	-	-	-
LOC439994	10	89102167	89103331	-	-	-	-	-
FAM22D	10	89117476	89130452	-	-	-	-0,001	-0,077
HTR7	10	92500575	92617671	-0,225	-0,054	-0,148	-0,246	0,032
RPP30	10	92631708	92668312	**-0,933**	-	-	-	-
ANKRD1	10	92671856	92681032	0,061	-	-	**-1,025**	-0,199
**13q14.2 *RB1***								
SUCLA2	13	48516790	48575462	-0,162	-	-	-	-
NUDT15	13	48611702	48621282	-0,075	-	-	-0,069	0,015
MED4	13	48649863	48669277	**-0,845**	-	-	-0,188	0,022
*MED4-AS1 **	13	48651272	48654129	-	-	-	-	-

INTS6	13	51353862	51454264	**-0,714**	-	-	-0,234	-0,165
**17p12 *MAP2K4***								
MYH13	17	10204182	10276322	0,028	-	-	-	-
MYH8	17	10293641	10325267	-0,018	-	-	-	-
MYH4	17	10346607	10372876	0,071	-	-	-	-
MYH1	17	10395626	10421859	0,073	-	-	-0,023	-0,062
MYH2	17	10424464	10452940	-0,121	-	-	-	-
MYH3	17	10531842	10560626	-0,142	-	-	-	-
SCO1	17	10583648	10600885	-0,301	-0,070	-0,102	0,100	**-0,734**
ELAC2	17	12894928	12921381	**-0,766**	-0,051	0,014	-0,770	0,177
**17q11.2 *NF1***								
GOSR1	17	28804425	28853832	-0,040	0,035	0,056	-	-
SUZ12P1	17	29036625	29097068	-	-	-	-	-
CRLF3	17	29109701	29151778	-0,094	-0,084	-0,127	-0,028	-0,063
ATAD5	17	29159022	29222295	-0,116	-	-	-0,092	0,004
TEFM	17	29226000	29233286	-0,352	-	-	-	-
ADAP2	17	29248753	29286211	-0,195	-	-	-	-
COPRS	17	30178883	30186326	-0,133	-	-	-	-
UTP6	17	30190189	30228729	-0,377	-	-	-	-
LRRC37B	17	30348154	30380519	-0,489	-	-	-	-
*SH3GL1P1 **	17	30367354	30369851	-	-	-	-	-
RHOT1	17	30469472	30552746	-0,122	-	-	-	-
*ARGFXP2 **	17	30477386	30478590	-	-	-	-	-
**18q21.2 *SMAD4***								
CCDC11	18	47753562	47792865	0,152	-	-	-0,027	0,022
MBD1	18	47793251	47808144	0,084	-0,050	0,101	-	-
CXXC1	18	47808712	47814692	-0,453	-	-	-0,535	-0,026
SKA1	18	47901391	47920538	-0,484	-0,301	-0,052	-	-

** non protein coding gene*

Supplementary Table 3

The sgRNA sequences used for fine-mapping essential genes in the *CDKN2A* region.

gene_id	UID	sgRNA nr	Strand	Start	End	5'-3'	5'-3'
ACER2	HGLibB_00430	1	+	19424717	19424736	CACCGCCATGCAACCCTTAGTTTCT	AAACAGAAACTAAGGGTTGCATGGC
ACER2	HGLibB_00431	2	-	19424729	19424748	CACCGGCATCTGACCCAAGAAACTA	AAACTAGTTTCTTGGGTCAGATGCC
ACER2	HGLibB_00432	3	-	19423894	19423913	CACCGACGAAACAAGCACATGCAGA	AAACTCTGCATGTGCTTGTTTCGTC
ACER2	HGLibA_00430	4	+	19409169	19409188	CACCGTATCGCCGAGTTCTACAACA	AAACTGTTGTAGAACTCGGCGATAC
ACER2	HGLibA_00431	5	-	19409177	19409196	CACCGCCGCACCGTGTTGTAGAACT	AAACAGTTCTACAACACGGTGCGGC
ACER2	HGLibA_00432	6	+	19423943	19423962	CACCGCTACTTAATCTGGACTCTTT	AAACAAAGAGTCCAGATTAAGTAGC
ACO1	HGLibA_00460	1	-	32419150	32419169	CACCGAATGGTGAGCACGATGTCAG	AAACCTGACATCGTGCTCACCATTC
ACO1	HGLibA_00461	2	-	32407393	32407412	CACCGATGACACGAGCAGGCTTAAA	AAACTTTAAGCCTGCTCGTGTCATC
ACO1	HGLibA_00462	3	+	32405519	32405538	CACCGCGCACACCTTGCTGAGCCAT	AAACATGGCTCAGCAAGGTGTGCGC
ACO1	HGLibB_00460	4	-	32407270	32407289	CACCGAGAAGAACTCTGATCGAAAA	AAACTTTTCGATCAGAGTTCTTCTC
ACO1	HGLibB_00461	5	-	32408603	32408622	CACCGTGATCTATTACAAGATCAGC	AAACGCTGATCTTGTAATAGATCAC
ACO1	HGLibB_00462	6	-	32408632	32408651	CACCGACCTTCTGTTGAAATCAACC	AAACGGTTGATTTCAACAGAAGGTC
ADAMTSL1	HGLibB_00845	1	+	18533234	18533253	CACCGTTGCAACTTAGGAGCTGTGA	AAACTCACAGCTCCTAAGTTGCAAC
ADAMTSL1	HGLibB_00846	2	-	18533269	18533288	CACCGCATTACTGCATGTTCTGTAT	AAACATACAGAACATGCAGTAATGC
ADAMTSL1	HGLibB_00847	3	-	18474245	18474264	CACCGGTGTGCCAGGAGTTGCCCGA	AAACTCGGGCAACTCCTGGCACACC
ADAMTSL1	HGLibA_00847	4	-	18504859	18504878	CACCGAGGCATCCCATAGGCCGTCC	AAACGGACGGCCTATGGGATGCCTC
ADAMTSL1	HGLibA_00848	5	-	18504834	18504853	CACCGCTCCTCGGAGCGTGCGGTCC	AAACGGACCGCACGCTCCGAGGAGC
ADAMTSL1	HGLibA_00849	6	+	18474226	18474245	CACCGCAAGCATGGAATGCTGCCGT	AAACACGGCAGCATTCCATGCTTGC
ANKRD18B	HGLibA_02020	1	-	33524502	33524521	CACCGCCAGGCGTCTCCCAAAACTG	AAACCAGTTTTGGGAGACGCCTGGC
ANKRD18B	HGLibA_02021	2	-	33524550	33524569	CACCGACCCCCGACCCGCATACTCT	AAACAGAGTATGCGGGTCGGGGGTC
ANKRD18B	HGLibA_02022	3	+	33529025	33529044	CACCGGCCATTATTCTCCTGAAACG	AAACCGTTTCAGGAGAATAATGGCC
ANKRD18B	HGLibB_02018	4	-	33528797	33528816	CACCGGTCTGTCACAGATGTTGATC	AAACGATCAACATCTGTGACAGACC
ANKRD18B	HGLibB_02019	5	+	33529090	33529109	CACCGCCATTATGCCGTGTATAATG	AAACCATTATACACGGCATAATGGC
APTX	HGLibA_02574	1	-	32974534	32974553	CACCGACATCTTCATGTGATCAGCC	AAACGGCTGATCACATGAAGATGTC
APTX	HGLibA_02575	2	-	32989875	32989894	CACCGGAGTGATGATGCGGGTGTGC	AAACGCACACCCGCATCATCACTCC
APTX	HGLibA_02576	3	-	32989759	32989778	CACCGAAGAAATGTTCTCGACAGCA	AAACTGCTGTCGAGAACATTTCTTC
APTX	HGLibB_02572	4	-	32988104	32988123	CACCGGTTGAAAGCAGAGTGTAACA	AAACTGTTACACTCTGCTTTCAACC
APTX	HGLibB_02573	5	+	32974537	32974556	CACCGTGATCACATGAAGATGTACA	AAACTGTACATCTTCATGTGATCAC
APTX	HGLibB_02574	6	-	32985986	32986005	CACCGAGGCTTGAAGATTTCTATGC	AAACGCATAGAAATCTTCAAGCCTC
AQP3	HGLibA_02592	1	-	33447472	33447491	CACCGAGATGCTCCACATCCGCTAC	AAACGTAGCGGATGTGGAGCATCTC
AQP3	HGLibA_02593	2	-	33447516	33447535	CACCGCCCCGCCATGGGTCGACAGA	AAACTCTGTCGACCCATGGCGGGGC
AQP3	HGLibA_02594	3	+	33443394	33443413	CACCGTGATCCAGGGCTCACGAGCC	AAACGGCTCGTGAGCCCTGGATCAC
AQP3	HGLibB_02590	4	+	33443845	33443864	CACCGTGCCCCGGCTGAGCACAACC	AAACGGTTGTGCTCAGCCGGGGCAC
AQP3	HGLibB_02591	5	-	33443376	33443395	CACCGCAAGCTGCCCATCTACACCC	AAACGGGTGTAGATGGGCAGCTTGC

AQP3	HGLibB_02592	6	-	33442887	33442906	CACCGATCTTTGCTACCTACCCCTC	AAACGAGGGGTAGGTAGCAAAGATC
AQP7	HGLibA_02604	1	+	33386493	33386512	CACCGGCCCAGCGCACAGTTAGCAA	AAACTTGCTAACTGTGCGCTGGGCC
AQP7	HGLibA_02605	2	-	33386498	33386517	CACCGGACCTTTGCTAACTGTGCGC	AAACGCGCACAGTTAGCAAAGGTCC
AQP7	HGLibA_02606	3	-	33395185	33395204	CACCGCACTGTCTTAGGTCCACCCG	AAACCGGGTGGACCTAAGACAGTGC
AQP7	HGLibB_02602	4	-	33386969	33386988	CACCGCACGTGGCAGGCCGCATCTC	AAACGAGATGCGGCCTGCCACGTGC
AQP7	HGLibB_02603	5	+	33395123	33395142	CACCGTCTTCCTCTGCAGTATTTCC	AAACGGAAATACTGCAGAGGAAGAC
AQP7	HGLibB_02604	6	+	33386977	33386996	CACCGGCCTGCCACGTGCACTCCCA	AAACTGGGAGTGCACGTGGCAGGCC
B4GALT1	HGLibA_04022	1	-	33120436	33120455	CACCGACGGCACATTTCCGTTGCAA	AAACTTGCAACGGAAATGTGCCGTC
B4GALT1	HGLibA_04023	2	+	33135266	33135285	CACCGTGCTCCTGCCGGTTGCGGAA	AAACTTCCGCAACCGGCAGGAGCAC
B4GALT1	HGLibA_04024	3	-	33120473	33120492	CACCGTGAATGACCATAATGCGTAC	AAACGTACGCATTATGGTCATTCAC
B4GALT1	HGLibB_04020	4	+	33135394	33135413	CACCGCATGTTAAACTCAATCAGCA	AAACTGCTGATTGAGTTTAACATGC
B4GALT1	HGLibB_04021	5	+	33113463	33113482	CACCGTCCCGATGTCCACTGTGATT	AAACAATCACAGTGGACATCGGGAC
B4GALT1	HGLibB_04022	6	-	33113562	33113581	CACCGTGACCGAATTGCACACACAA	AAACTTGTGTGTGCAATTCGGTCAC
BAG1	HGLibA_04073	1	-	33255865	33255884	CACCGAGGCTTGGTAAAAAAGGTTC	AAACGAACCTTTTTTACCAAGCCTC
BAG1	HGLibA_04074	2	-	33262763	33262782	CACCGTGAACCAGTTGTCCAAGACC	AAACGGTCTTGGACAACTGGTTCAC
BAG1	HGLibA_04075	3	-	33261095	33261114	CACCGGGTTGCCGGGTCATGTTAAT	AAACATTAACATGACCCGGCAACCC
BAG1	HGLibB_04071	4	+	33262736	33262755	CACCGCTATGACCTCTTCAACAACC	AAACGGTTGTTGAAGAGGTCATAGC
BAG1	HGLibB_04072	5	-	33261128	33261147	CACCGGAAACACCGTTGTCAGCACT	AAACAGTGCTGACAACGGTGTTTCC
BAG1	HGLibB_04073	6	-	33258956	33258975	CACCGGGAGAAGATAGCTGACCAGC	AAACGCTGGTCAGCTATCTTCTCCC
BNC2	HGLibA_04646	1	-	16727856	16727875	CACCGAGAACGACTACGGCTGAACC	AAACGGTTCAGCCGTAGTCGTTCTC
BNC2	HGLibA_04647	2	+	16727870	16727889	CACCGCGTTCTGGTTCCGAACTGCA	AAACTGCAGTTCGGAACCAGAACGC
BNC2	HGLibA_04648	3	-	16583068	16583087	CACCGTCAGGCCATCCGTTGCACAC	AAACGTGTGCAACGGATGGCCTGAC
BNC2	HGLibB_04644	4	+	16552681	16552700	CACCGGCTGATGTCAAACACGACGT	AAACACGTCGTGTTTGACATCAGCC
BNC2	HGLibB_04645	5	+	16552611	16552630	CACCGCGCTGAAGAGACGGTCCAGC	AAACGCTGGACCGTCTCTTCAGCGC
BNC2	HGLibB_04646	6	+	16583002	16583021	CACCGTACACTGATCACAAGTCCTC	AAACGAGGACTTGTGATCAGTGTAC
C9orf72	HGLibA_06757	1	-	27566790	27566809	CACCGAATGGGGATCGCAGCACATA	AAACTATGTGCTGCGATCCCCATTC
C9orf72	HGLibA_06758	2	-	27558491	27558510	CACCGCTTTGTACAAGGCCTGCTAA	AAACTTAGCAGGCCTTGTACAAAGC
C9orf72	HGLibA_06759	3	-	27562449	27562468	CACCGAGTATTATTCCAATGCTTAC	AAACGTAAGCATTGGAATAATACTC
C9orf72	HGLibB_06752	4	+	27562437	27562456	CACCGATCACTTCTCCAGTAAGCAT	AAACATGCTTACTGGAGAAGTGATC
C9orf72	HGLibB_06753	5	+	27566884	27566903	CACCGCACCACTCTCTGCATTTCGA	AAACTCGAAATGCAGAGAGTGGTGC
C9orf72	HGLibB_06754	6	+	27558534	27558553	CACCGTGATTCTGCTTCACATAACC	AAACGGTTATGTGAAGCAGAATCAC
CAAP1	HGLibA_06829	1	-	26887354	26887373	CACCGCAGCAGTGCTTCTGTATTAT	AAACATAATACAGAAGCACTGCTGC
CAAP1	HGLibA_06830	2	+	26887303	26887322	CACCGTGAAGTACCTTTAACACATC	AAACGATGTGTTAAAGGTACTTCAC
CAAP1	HGLibA_06831	3	-	26884872	26884891	CACCGTTTATAAGGTGACAATGGAA	AAACTTCCATTGTCACCTTATAAAC
CAAP1	HGLibB_06824	4	multiple	26861084	26861103	CACCGAGAGAGAATAAGCAACCTGA	AAACTCAGGTTGCTTATTCTCTCTC
CAAP1	HGLibB_06825	5	-	26884841	26884860	CACCGATGGAAGAGGAAGCAGATGA	AAACTCATCTGCTTCCTCTTCCATC
CAAP1	HGLibB_06826	6	-	26886153	26886172	CACCGAGAAATTAAAAAACTATGCC	AAACGGCATAGTTTTTTAATTTCTC
CDKN2A	HGLibA_08829	1	+	21974684	21974703	CACCGGGCCTCCGACCGTAACTATT	AAACAATAGTTACGGTCGGAGGCCC
CDKN2A	HGLibA_08830	2	+	21994282	21994301	CACCGGGCCGCACGCGCGCCGAATC	AAACGATTCGGCGCGCGTGCGGCCC
CDKN2A	HGLibA_08831	3	-	21971100	21971119	CACCGGACCCGTGCACGACGCTGCC	AAACGGCAGCGTCGTGCACGGGTCC
CDKN2A	HGLibB_08822	4	-	21994298	21994317	CACCGTCTTGGTGACCCTCCGGATT	AAACAATCCGGAGGGTCACCAAGAC

CDKN2A	HGLibB_08823	5	-	21974696	21974715	CACCGCCCAACGCACCGAATAGTTA	AAACTAACTATTCGGTGCGTTGGGC
CDKN2A	HGLibB_08824	6	-	21968726	21968745	CACCGTTGTAGAAGCAGGCATGCGT	AAACACGCATGCCTGCTTCTACAAC
CDKN2B	HGLibA_08838	1	+	22008799	22008818	CACCGGGATCGCGCGCCTCCCGAAA	AAACTTTCGGGAGGCGCGCGATCCC
CDKN2B	HGLibA_08839	2	+	22006006	22006025	CACCGGCAGGTACCCTGCAACGTCG	AAACCGACGTTGCAGGGTACCTGCC
CDKN2B	HGLibA_08840	3	+	22008824	22008843	CACCGGACTCCGTTGGGATCCGCGC	AAACGCGCGGATCCCAACGGAGTCC
CDKN2B	HGLibB_08831	4	-	22006017	22006036	CACCGCGGGGCCACCGCGACGTTGC	AAACGCAACGTCGCGGTGGCCCCGC
CDKN2B	HGLibB_08832	5	-	22008816	22008835	CACCGCCCAACGGAGTCAACCGTTT	AAACAAACGGTTGACTCCGTTGGGC
CDKN2B	HGLibB_08833	6	+	22008869	22008888	CACCGCTCCACTAGTCCCCGCGCCG	AAACCGGCGCGGGGACTAGTGGAGC
CHMP5	HGLibA_09495	1	+	33270678	33270697	CACCGTTATACCATCCAGTCTTTGA	AAACTCAAAGACTGGATGGTATAAC
CHMP5	HGLibA_09496	2	+	33270645	33270664	CACCGTGCCCAACAGTCATTCAACA	AAACTGTTGAATGACTGTTGGGCAC
CHMP5	HGLibA_09497	3	-	33276535	33276554	CACCGAAATCATCTTCATCCAGTTC	AAACGAACTGGATGAAGATGATTTC
CHMP5	HGLibB_09486	4	+	33276541	33276560	CACCGGATGAAGATGATTTAGAAGC	AAACGCTTCTAAATCATCTTCATCC
CHMP5	HGLibB_09487	5	-	33266030	33266049	CACCGTCGAGAAATCTTCTTGTCAA	AAACTTGACAAGAAGATTTCTCGAC
CHMP5	HGLibB_09488	6	+	33266031	33266050	CACCGTGACAAGAAGATTTCTCGAT	AAACATCGAGAAATCTTCTTGTCAC
CNTLN	HGLibA_10397	1	+	17135153	17135172	CACCGAGTACACGCAATGCGCAGCG	AAACCGCTGCGCATTGCGTGTACTC
CNTLN	HGLibA_10398	2	+	17135096	17135115	CACCGTTCGCCCCCAGCGCGACAGC	AAACGCTGTCGCGCTGGGGGCGAAC
CNTLN	HGLibA_10399	3	-	17143340	17143359	CACCGCTGACCACTTGTGTGAGATC	AAACGATCTCACACAAGTGGTCAGC
CNTLN	HGLibB_10386	4	+	17143333	17143352	CACCGAAACCCAGATCTCACACAAG	AAACCTTGTGTGAGATCTGGGTTTC
CNTLN	HGLibB_10387	5	+	17226237	17226256	CACCGTCTAGAAATTCTGCAAGTCA	AAACTGACTTGCAGAATTTCTAGAC
CNTLN	HGLibB_10388	6	+	17226264	17226283	CACCGCAAAATACAAGAATTTGAAC	AAACGTTCAAATTCTTGTATTTTGC
DDX58	HGLibA_12752	1	+	32526107	32526126	CACCGGGGTCTTCCGGATATAATCC	AAACGGATTATATCCGGAAGACCCC
DDX58	HGLibA_12753	2	+	32526135	32526154	CACCGTTGCAGGCTGCGTCGCTGCT	AAACAGCAGCGACGCAGCCTGCAAC
DDX58	HGLibA_12754	3	-	32492524	32492543	CACCGTGCTTAGATTTGCTCTACTA	AAACTAGTAGAGCAAATCTAAGCAC
DDX58	HGLibB_12738	4	-	32492470	32492489	CACCGATGCCTTCTCAGATCAGACA	AAACTGTCTGATCTGAGAAGGCATC
DDX58	HGLibB_12739	5	-	32493782	32493801	CACCGGTCTGAATGTTTAATTAATC	AAACGATTAATTAAACATTCAGACC
DDX58	HGLibB_12740	6	+	32493897	32493916	CACCGTTTGAAATCCCAACTTTCAA	AAACTTGAAAGTTGGGATTTCAAAC
DENND4C	HGLibB_12937	1	+	19276383	19276402	CACCGCTCCAAGCAAACTTGAACTA	AAACTAGTTCAAGTTTGCTTGGAGC
DENND4C	HGLibB_12938	2	-	19288604	19288623	CACCGCTTATAACACAGAAACACGC	AAACGCGTGTTTCTGTGTTATAAGC
DENND4C	HGLibB_12939	3	-	19288636	19288655	CACCGTATGCTATTGCATTTGAAGC	AAACGCTTCAAATGCAATAGCATAC
DENND4C	HGLibA_12955	4	+	19276200	19276219	CACCGACAGACTACTTTGTCGTAGC	AAACGCTACGACAAAGTAGTCTGTC
DENND4C	HGLibA_12956	5	+	19286810	19286829	CACCGGTGATCCTAGCCACACCCTA	AAACTAGGGTGTGGCTAGGATCACC
DENND4C	HGLibA_12957	6	-	19286818	19286837	CACCGAGCGACCATAGGGTGTGGCT	AAACAGCCACACCCTATGGTCGCTC
DMRTA1	HGLibA_13425	1	-	22447097	22447116	CACCGGAGGTCGGCCGCTAACGCCT	AAACAGGCGTTAGCGGCCGACCTCC
DMRTA1	HGLibA_13426	2	+	22451671	22451690	CACCGTACGGCGTAAATCCTAGAGT	AAACACTCTAGGATTTACGCCGTAC
DMRTA1	HGLibA_13427	3	-	22447156	22447175	CACCGTACCGGCAACGCCGGGGACG	AAACCGTCCCCGGCGTTGCCGGTAC
DMRTA1	HGLibB_13406	4	+	22451322	22451341	CACCGCAAAGACTTGACTGCGACCA	AAACTGGTCGCAGTCAAGTCTTTGC
DMRTA1	HGLibB_13407	5	-	22447456	22447475	CACCGGGCCATGACGCGCTGGCGCT	AAACAGCGCCAGCGCGTCATGGCCC
DMRTA1	HGLibB_13408	6	-	22447690	22447709	CACCGTTGCTGGAAAACTTCGAAAG	AAACCTTTCGAAGTTTTCCAGCAAC
DNAJA1	HGLibA_13509	1	+	33036606	33036625	CACCGCAGCTCGTTGAAGCACTGTG	AAACCACAGTGCTTCAACGAGCTGC
DNAJA1	HGLibA_13510	2	-	33036679	33036698	CACCGAGTTCCCATACCTGGATGAG	AAACCTCATCCAGGTATGGGAACTC
DNAJA1	HGLibA_13511	3	+	33030464	33030483	CACCGGAGTGCTGTCCCAATTGCCG	AAACCGGCAATTGGGACAGCACTCC

DNAJA1	HGLibB_13490	4	+	33030644	33030663	CACCGTTAGAAGTTCATATTGACAA	AAACTTGTCAATATGAACTTCTAAC
DNAJA1	HGLibB_13491	5	+	33026867	33026886	CACCGATATGACAAAGGAGGAGAAC	AAACGTTCTCCTCCTTTGTCATATC
DNAJA1	HGLibB_13492	6	-	33029917	33029936	CACCGCACCATTATATAAGTCTTCT	AAACAGAAGACTTATATAATGGTGC
ELAVL2	HGLibA_14881	1	-	23701393	23701412	CACCGTCCAGGACCGCTAGCTCAGC	AAACGCTGAGCTAGCGGTCCTGGAC
ELAVL2	HGLibA_14882	2	-	23704925	23704944	CACCGTTCTCGTATTCTTGTCGACC	AAACGGTCGACAAGAATACGAGAAC
ELAVL2	HGLibA_14883	3	-	23701560	23701579	CACCGTCGATTTGACAAGCGAATTG	AAACCAATTCGCTTGTCAAATCGAC
ELAVL2	HGLibB_14859	4	-	23731082	23731101	CACCGTGTGAACTACATTGACCCCA	AAACTGGGGTCAATGTAGTTCACAC
ELAVL2	HGLibB_14860	5	-	23693465	23693484	CACCGGTTGGACAATCTGCTCAATA	AAACTATTGAGCAGATTGTCCAACC
ELAVL2	HGLibB_14861	6	+	23731034	23731053	CACCGTTTGAAGTCTCAATCCATTC	AAACGAATGGATTGAGACTTCAAAC
EQTN	HGLibA_15397	1	+	27296968	27296987	CACCGGCTCTCTCACCTTCAATAGT	AAACACTATTGAAGGTGAGAGAGCC
EQTN	HGLibA_15398	2	-	27296980	27296999	CACCGACTTTGAAGCCTACTATTGA	AAACTCAATAGTAGGCTTCAAAGTC
EQTN	HGLibA_15399	3	-	27296640	27296659	CACCGGCTCCTGCTAATGAGAAAAA	AAACTTTTTCTCATTAGCAGGAGCC
EQTN	HGLibB_15375	4	+	27294358	27294377	CACCGATTTCAGACTCAGTGCCATT	AAACAATGGCACTGAGTCTGAAATC
EQTN	HGLibB_15376	5	-	27296695	27296714	CACCGAAATGAAGATGTTAATAAGC	AAACGCTTATTAACATCTTCATTTC
EQTN	HGLibB_15377	6	-	27294376	27294395	CACCGTTCACAACACAAAATCCAAA	AAACTTTGGATTTTGTGTTGTGAAC
FAM154A	HGLibB_16151	1	+	18928282	18928301	CACCGCCTCGAGGGCCAGACCAATG	AAACCATTGGTCTGGCCCTCGAGGC
FAM154A	HGLibB_16152	2	+	18950748	18950767	CACCGGCCCTCACCTTGATGTAGTC	AAACGACTACATCAAGGTGAGGGCC
FAM154A	HGLibB_16153	3	+	18950775	18950794	CACCGCCATTGGTATAGGCCCTTTC	AAACGAAAGGGCCTATACCAATGGC
FAM154A	HGLibA_16173	4	+	18941654	18941673	CACCGCACTCCATCTTGTCGCTACA	AAACTGTAGCGACAAGATGGAGTGC
FAM154A	HGLibA_16174	5	-	18941659	18941678	CACCGATATCCATGTAGCGACAAGA	AAACTCTTGTCGCTACATGGATATC
FAM154A	HGLibA_16175	6	-	18928904	18928923	CACCGGACCATAAGCTGTAAACCTC	AAACGAGGTTTACAGCTTATGGTCC
FOCAD	HGLibA_17776	1	+	20717846	20717865	CACCGGATTCACCAATCTACAAATC	AAACGATTTGTAGATTGGTGAATCC
FOCAD	HGLibA_17777	2	+	20717804	20717823	CACCGTCTTATTGCTGCAGTCCTAA	AAACTTAGGACTGCAGCAATAAGAC
FOCAD	HGLibA_17778	3	+	20740318	20740337	CACCGTTCAGAGTATATATACCATT	AAACAATGGTATATATACTCTGAAC
FOCAD	HGLibB_17753	4	-	20770132	20770151	CACCGACAGCAGCTGAAGACTCATC	AAACGATGAGTCTTCAGCTGCTGTC
FOCAD	HGLibB_17754	5	-	20770207	20770226	CACCGGTTCAACACTGTGCTCTAAA	AAACTTTAGAGCACAGTGTTGAACC
FOCAD	HGLibB_17755	6	+	20740271	20740290	CACCGACTACAAATGCAAGCTCTTA	AAACTAAGAGCTTGCATTTGTAGTC
HAUS6	HGLibB_20860	1	-	19093270	19093289	CACCGAGTAGCTTTCCTCAAGTTGT	AAACACAACTTGAGGAAAGCTACTC
HAUS6	HGLibB_20861	2	-	19093246	19093265	CACCGTCACTATTTCTTTCTCCTGG	AAACCCAGGAGAAAGAAATAGTGAC
HAUS6	HGLibB_20862	3	-	19089423	19089442	CACCGTTGTGTTACCCAAAAATATC	AAACGATATTTTTGGGTAACACAAC
HAUS6	HGLibA_20888	4	+	19089465	19089484	CACCGAAAATCTGCTACGTGCGAAA	AAACTTTCGCACGTAGCAGATTTTC
HAUS6	HGLibA_20889	5	+	19089497	19089516	CACCGATGCATTTGTGCAAGTCCTG	AAACCAGGACTTGCACAAATGCATC
HAUS6	HGLibA_20890	6	-	19094333	19094352	CACCGTCCGAAAACATTGCTGTGAA	AAACTTCACAGCAATGTTTTCGGAC
IFNA10	HGLibA_22658	1	-	21206910	21206929	CACCGACATGATTTCCGAATCCCCC	AAACGGGGGATTCGGAAATCATGTC
IFNA10	HGLibA_22659	2	-	21206629	21206648	CACCGTCACTCTTTATCTAATAGAG	AAACCTCTATTAGATAAAGAGTGAC
IFNA10	HGLibA_22660	3	+	21206955	21206974	CACCGAGATTCTTCCCATTTGTCCC	AAACGGGACAAATGGGAAGAATCTC
IFNA10	HGLibB_22627	4	+	21206919	21206938	CACCGGGAAATCATGTCTGTCCTTC	AAACGAAGGACAGACATGATTTCCC
IFNA10	HGLibB_22628	5	+	21207069	21207088	CACCGCCATAAGTAAAGAAAAGGAC	AAACGTCCTTTTCTTTACTTATGGC
IFNA14	HGLibA_22664	1	+	21239820	21239839	CACCGTCAAAGTCCTCCTGTTATTC	AAACGAATAACAGGAGGACTTTGAC
IFNA14	HGLibA_22665	2	-	21239833	21239852	CACCGAAACCCACAGCCTGAATAAC	AAACGTTATTCAGGCTGTGGGTTTC
IFNA14	HGLibA_22666	3	+	21239727	21239746	CACCGGGTTGCCATCAAATTCCTCC	AAACGGAGGAATTTGATGGCAACCC

IFNA14	HGLibB_22633	4	multiple	21239551	21239570	CACCGCCCAACCTCCTGTATCACAC	AAACGTGTGATACAGGAGGTTGGGC
IFNA14	HGLibB_22634	5	-	21239580	21239599	CACCGTTTCCAGCAAATGAATGACC	AAACGGTCATTCATTTGCTGGAAAC
IFNA14	HGLibB_22635	6	+	21239574	21239593	CACCGCTTCCAGGTCATTCATTTGC	AAACGCAAATGAATGACCTGGAAGC
IFNA16	HGLibA_22667	1	+	21216921	21216940	CACCGCCCAACCTCCTGTGTCACAC	AAACGTGTGACACAGGAGGTTGGGC
IFNA16	HGLibA_22668	2	multiple	21217047	21217066	CACCGCTGCTGGATCATCTCATGGA	AAACTCCATGAGATGATCCAGCAGC
IFNA16	HGLibA_22669	3	-	21217118	21217137	CACCGATATGATTTCGGATTCCCCC	AAACGGGGGAATCCGAAATCATATC
IFNA16	HGLibB_22636	4	-	21216932	21216951	CACCGCCTAGAAGCCTGTGTGACAC	AAACGTGTCACACAGGCTTCTAGGC
IFNA16	HGLibB_22637	5	+	21216971	21216990	CACCGGTTCAATGTAGAATTTGTCT	AAACAGACAAATTCTACATTGAACC
IFNA16	HGLibB_22638	6	+	21217097	21217116	CACCGGGTTGCCATCAAACACCTCC	AAACGGAGGTGTTTGATGGCAACCC
IFNA17	HGLibA_22670	1	-	21227986	21228005	CACCGACATGACTTTGGACTTCCCC	AAACGGGGAAGTCCAAAGTCATGTC
IFNA17	HGLibA_22671	2	-	21227818	21227837	CACCGTTACCAGCAACTGAATAACC	AAACGGTTATTCAGTTGCTGGTAAC
IFNA17	HGLibA_22672	3	-	21227618	21227637	CACCGCAAACTTGCAAAAAATATTA	AAACTAATATTTTTTGCAAGTTTGC
IFNA2	HGLibA_22673	1	-	21385307	21385326	CACCGGGCCTTGACCTTTGCTTTAC	AAACGTAAAGCAAAGGTCAAGGCCC
IFNA2	HGLibA_22674	2	-	21385115	21385134	CACCGGTTTGGCAACCAGTTCCAAA	AAACTTTGGAACTGGTTGCCAAACC
IFNA2	HGLibA_22675	3	+	21385121	21385140	CACCGACTGGTTGCCAAACTCCTCC	AAACGGAGGAGTTTGGCAACCAGTC
IFNA2	HGLibB_22642	4	+	21385302	21385321	CACCGCACCAGTAAAGCAAAGGTCA	AAACTGACCTTTGCTTTACTGGTGC
IFNA2	HGLibB_22643	5	+	21385214	21385233	CACCGTCAAGGTCCTCCTGCTACCC	AAACGGGTAGCAGGAGGACCTTGAC
IFNA2	HGLibB_22644	6	-	21384926	21384945	CACCGGACAGAGACTCCCCTGATGA	AAACTCATCAGGGGAGTCTCTGTCC
IFNA21	HGLibA_22676	1	+	21166228	21166247	CACCGCCCAACCTCCTGTATCACGC	AAACGCGTGATACAGGAGGTTGGGC
IFNA21	HGLibA_22677	2	-	21166425	21166444	CACCGACATGACTTTGGATTCCCCC	AAACGGGGGAATCCAAAGTCATGTC
IFNA21	HGLibA_22678	3	-	21166232	21166251	CACCGGCCTGCGTGATACAGGAGGT	AAACACCTCCTGTATCACGCAGGCC
IFNA21	HGLibB_22645	4	-	21166257	21166276	CACCGTAACCAGCAGCTGAATGACC	AAACGGTCATTCAGCTGCTGGTTAC
IFNA4	HGLibA_22679	1	-	21187344	21187363	CACCGACATGATTTCGGATTCCCCG	AAACCGGGGAATCCGAAATCATGTC
IFNA4	HGLibA_22680	2	+	21187353	21187372	CACCGCGAAATCATGTCTGTCCTTC	AAACGAAGGACAGACATGATTTCGC
IFNA4	HGLibB_22648	4	multiple	21187314	21187333	CACCGTGATGGCCACCAGTTCCAGA	AAACTCTGGAACTGGTGGCCATCAC
IFNA5	HGLibA_22682	1	+	21304872	21304891	CACCGTCCAACCTCCTGCATCATAC	AAACGTATGATGCAGGAGGTTGGAC
IFNA5	HGLibA_22683	2	-	21305192	21305211	CACCGCTGCAAGTCAATCTGTTCTC	AAACGAGAACAGATTGACTTGCAGC
IFNA5	HGLibA_22684	3	-	21305154	21305173	CACCGAGACCCACAGCCTGAGTAAC	AAACGTTACTCAGGCTGTGGGTCTC
IFNA5	HGLibB_22651	4	-	21305126	21305145	CACCGTTTGATGATAATGGCACAAA	AAACTTTGTGCCATTATCATCAAAC
IFNA5	HGLibB_22652	5	multiple	21304781	21304800	CACCGATTTCTTCTCTGTCAGATAG	AAACCTATCTGACAGAGAAGAAATC
IFNA5	HGLibB_22653	6	+	21305141	21305160	CACCGTCAAAGTCCTCCTGTTACTC	AAACGAGTAACAGGAGGACTTTGAC
IFNA6	HGLibA_22685	1	+	21350772	21350791	CACCGTCATGGTCCTCCTGTGACCC	AAACGGGTCACAGGAGGACCATGAC
IFNA6	HGLibA_22686	2	-	21350581	21350600	CACCGCATCTGTTGCTTGGGATGAG	AAACCTCATCCCAAGCAACAGATGC
IFNA6	HGLibA_22687	3	-	21350700	21350719	CACCGACATGACTTCAGATTTCCCC	AAACGGGGAAATCTGAAGTCATGTC
IFNA6	HGLibB_22654	4	+	21350625	21350644	CACCGAGGTCTGCTGAATCACCTCA	AAACTGAGGTGATTCAGCAGACCTC
IFNA6	HGLibB_22655	5	-	21350338	21350357	CACCGCATCAAGAAACTTGCAAGAA	AAACTTCTTGCAAGTTTCTTGATGC
IFNA6	HGLibB_22656	6	multiple	21350643	21350662	CACCGAGCCATCTCTGTCCTCCATG	AAACCATGGAGGACAGAGATGGCTC
IFNA7	HGLibA_22688	1	-	21202063	21202082	CACCGAGACCCACAGCCTGCGTAAT	AAACATTACGCAGGCTGTGGGTCTC
IFNA7	HGLibA_22689	2	-	21201978	21201997	CACCGACATGAATTCAGATTCCCAG	AAACCTGGGAATCTGAATTCATGTC
IFNA7	HGLibA_22690	3	+	21202050	21202069	CACCGTCAAGGCCCTCCTATTACGC	AAACGCGTAATAGGAGGGCCTTGAC
IFNA7	HGLibB_22657	4	-	21202101	21202120	CACCGCTACAAATCCATCTGCTCTC	AAACGAGAGCAGATGGATTTGTAGC

IFNA7	HGLibB_22658	5	+	21201991	21202010	CACCGTTCATGTCTGTCCTTCAAGC	AAACGCTTGAAGGACAGACATGAAC
IFNA7	HGLibB_22659	6	+	21202090	21202109	CACCGATCACAGCCCAGAGAGCAGA	AAACTCTGCTCTCTGGGCTGTGATC
IFNA8	HGLibA_22691	1	+	21409565	21409584	CACCGAGAGTCTCCCCTGATGTACG	AAACCGTACATCAGGGGAGACTCTC
IFNA8	HGLibA_22692	2	-	21409487	21409506	CACCGCGATGTAGAATTCATCTAGA	AAACTCTAGATGAATTCTACATCGC
IFNA8	HGLibA_22693	3	-	21409208	21409227	CACCGACTTGTAGCTGAGCACCACT	AAACAGTGGTGCTCAGCTACAAGTC
IFNA8	HGLibB_22660	4	-	21409364	21409383	CACCGGTTTATCATCAAACTCCTCC	AAACGGAGGAGTTTGATGATAAACC
IFNA8	HGLibB_22661	5	+	21409373	21409392	CACCGTGATGATAAACAGTTCCAGA	AAACTCTGGAACTGTTTATCATCAC
IFNA8	HGLibB_22662	6	+	21409535	21409554	CACCGGTCCTGTGTGATGCAGGAAG	AAACCTTCCTGCATCACACAGGACC
IFNB1	HGLibA_22700	1	+	21077295	21077314	CACCGTAGGAGATCTTCAGTTTCGG	AAACCCGAAACTGAAGATCTCCTAC
IFNB1	HGLibA_22701	2	-	21077789	21077808	CACCGTCCATGAGCTACAACTTGCT	AAACAGCAAGTTGTAGCTCATGGAC
IFNB1	HGLibA_22702	3	+	21077724	21077743	CACCGGCCTCCCATTCAATTGCCAC	AAACGTGGCAATTGAATGGGAGGCC
IFNB1	HGLibB_22669	4	-	21077544	21077563	CACCGGACTATTGTTGAGAACCTCC	AAACGGAGGTTCTCAACAATAGTCC
IFNB1	HGLibB_22670	5	+	21077359	21077378	CACCGTTCCACTCTGACTATGGTCC	AAACGGACCATAGTCAGAGTGGAAC
IFNB1	HGLibB_22671	6	+	21077523	21077542	CACCGTCTGATGATAGACATTAGCC	AAACGGCTAATGTCTATCATCAGAC
IFNE	HGLibA_22703	1	-	21481073	21481092	CACCGCTACAGAGTTTAGAAGCCCG	AAACCGGGCTTCTAAACTCTGTAGC
IFNE	HGLibA_22704	2	+	21481097	21481116	CACCGCTCTTGCTTCATGTCGTTCA	AAACTGAACGACATGAAGCAAGAGC
IFNE	HGLibA_22705	3	+	21481315	21481334	CACCGCCAGTCCCATGAGTGCTTCT	AAACAGAAGCACTCATGGGACTGGC
IFNE	HGLibB_22672	4	+	21481181	21481200	CACCGTTCTACTTGGACAATGGCCC	AAACGGGCCATTGTCCAAGTAGAAC
IFNE	HGLibB_22673	5	-	21481324	21481343	CACCGAGAATACCTAGAAGCACTCA	AAACTGAGTGCTTCTAGGTATTCTC
IFNE	HGLibB_22674	6	-	21481247	21481266	CACCGAAGTTAAAATGTACTTCCGA	AAACTCGGAAGTACATTTTAACTTC
IFNK	HGLibA_22715	1	-	27524418	27524437	CACCGCGTTCAGTAAGTTACAGTCC	AAACGGACTGTAACTTACTGAACGC
IFNK	HGLibA_22716	2	-	27524679	27524698	CACCGCTTGCTGATCAAGTCCTATT	AAACAATAGGACTTGATCAGCAAGC
IFNK	HGLibA_22717	3	+	27524383	27524402	CACCGCTTATGGGTATATTCATTGC	AAACGCAATGAATATACCCATAAGC
IFNK	HGLibB_22684	4	+	27524438	27524457	CACCGTTCACCTGAGAAGAGTCACC	AAACGGTGACTCTTCTCAGGTGAAC
IFNK	HGLibB_22685	5	-	27524385	27524404	CACCGCAGCAATGAATATACCCATA	AAACTATGGGTATATTCATTGCTGC
IFNK	HGLibB_22686	6	+	27524700	27524719	CACCGAGAGTACCTGAACCAATGCT	AAACAGCATTGGTTCAGGTACTCTC
IFNW1	HGLibA_22733	1	+	21141519	21141538	CACCGGCTATAGCTGGTCATCACTA	AAACTAGTGATGACCAGCTATAGCC
IFNW1	HGLibA_22734	2	-	21141514	21141533	CACCGATGACCAGCTATAGCCCTGT	AAACACAGGGCTATAGCTGGTCATC
IFNW1	HGLibA_22735	3	+	21141438	21141457	CACCGCATTTGGTGCAGAAGCACCA	AAACTGGTGCTTCTGCACCAAATGC
IFNW1	HGLibB_22702	4	-	21141191	21141210	CACCGGACCTGCTTGCTGCAGGTAG	AAACCTACCTGCAGCAAGCAGGTCC
IFNW1	HGLibB_22703	5	+	21141186	21141205	CACCGTCCCACTACCTGCAGCAAGC	AAACGCTTGCTGCAGGTAGTGGGAC
IFNW1	HGLibB_22704	6	-	21141141	21141160	CACCGGCAGCCCTGCACTGACCTTG	AAACCAAGGTCAGTGCAGGGCTGCC
IFT74	HGLibA_22769	1	+	26961990	26962009	CACCGGCAGCTCGCCCTGTTTCAAG	AAACCTTGAAACAGGGCGAGCTGCC
IFT74	HGLibA_22770	2	+	26962055	26962074	CACCGCCTATCAGGAAATATTCGAG	AAACCTCGAATATTTCCTGATAGGC
IFT74	HGLibA_22771	3	+	26980586	26980605	CACCGTTAGACAAATCTTACTATCT	AAACAGATAGTAAGATTTGTCTAAC
IFT74	HGLibB_22738	4	-	26980576	26980595	CACCGATTTGTCTAAAATTTGCCTC	AAACGAGGCAAATTTTAGACAAATC
IFT74	HGLibB_22739	5	+	26984279	26984298	CACCGTGAAGTTAATAAACTTCAGA	AAACTCTGAAGTTTATTAACTTCAC
IFT74	HGLibB_22740	6	+	26984332	26984351	CACCGTATATTTGTCATATGAAAAG	AAACCTTTTCATATGACAAATATAC
IZUMO3	HGLibA_23897	1	-	24545665	24545684	CACCGGACAACCTGCTGTCGGGGAG	AAACCTCCCCGACAGCAGGTTGTCC
IZUMO3	HGLibA_23898	2	-	24545482	24545501	CACCGGCTGCTTGAACGGCAGATTA	AAACTAATCTGCCGTTCAAGCAGCC
IZUMO3	HGLibA_23899	3	-	24545276	24545295	CACCGTCCCTCTCTAGCTGTTCAGC	AAACGCTGAACAGCTAGAGAGGGAC

IZUMO3	HGLibB_23863	4	-	24545015	24545034	CACCGTCTCGATGTCTGCCAAAACC	AAACGGTTTTGGCAGACATCGAGAC
IZUMO3	HGLibB_23864	5	-	24545044	24545063	CACCGTTAGGTGTCTTTATCTATCA	AAACTGATAGATAAAGACACCTAAC
IZUMO3	HGLibB_23865	6	+	24545488	24545507	CACCGGCCGTTCAAGCAGCTGAGTT	AAACAACTCAGCTGCTTGAACGGCC
KLHL9	HGLibA_25227	1	+	21334825	21334844	CACCGCGCCCATTTCGCCGTTACCA	AAACTGGTAACGGCGAAATGGGCGC
KLHL9	HGLibA_25228	2	-	21333840	21333859	CACCGGATGCTCCCCGTTACCAGCA	AAACTGCTGGTAACGGGGAGCATCC
KLHL9	HGLibA_25229	3	+	21334181	21334200	CACCGTTCCAACCTTAGCCAGCGAC	AAACGTCGCTGGCTAAGGTTGGAAC
KLHL9	HGLibB_25192	4	+	21333717	21333736	CACCGTGCATCCATTTATTATACCG	AAACCGGTATAATAAATGGATGCAC
KLHL9	HGLibB_25193	5	-	21334354	21334373	CACCGCACCTACAATCTTATAGAAG	AAACCTTCTATAAGATTGTAGGTGC
KLHL9	HGLibB_25194	6	-	21334186	21334205	CACCGAGCCTGTCGCTGGCTAAGGT	AAACACCTTAGCCAGCGACAGGCTC
LINGO2	HGLibA_26587	1	-	27950512	27950531	CACCGCGATTGATCGCCATCCCAGA	AAACTCTGGGATGGCGATCAATCGC
LINGO2	HGLibA_26588	2	+	27949320	27949339	CACCGACGCCTTCGGGGTGTCACCC	AAACGGGTGACACCCCGAAGGCGTC
LINGO2	HGLibA_26589	3	-	27950306	27950325	CACCGTCGTCTAAAGCTGGTCCCTT	AAACAAGGGACCAGCTTTAGACGAC
LINGO2	HGLibB_26550	4	-	27949250	27949269	CACCGCTTGGAAATCCGCTTTGCCC	AAACGGGCAAAGCGGATTTCCAAGC
LINGO2	HGLibB_26551	5	+	27950287	27950306	CACCGAGCCCCGTGAATACTCCCAA	AAACTTGGGAGTATTCACGGGGCTC
LINGO2	HGLibB_26552	6	+	27949913	27949932	CACCGATGTGAGGTTGAGACCGTAG	AAACCTACGGTCTCAACCTCACATC
LRRC19	HGLibA_27274	1	-	26995710	26995729	CACCGTAGTTATAATCATCATCGCC	AAACGGCGATGATGATTATAACTAC
LRRC19	HGLibA_27275	2	-	26998043	26998062	CACCGATCTTACATAATAACGGTTT	AAACAAACCGTTATTATGTAAGATC
LRRC19	HGLibA_27276	3	-	26997981	26998000	CACCGCTCCATCTATGTAATTCAAC	AAACGTTGAATTACATAGATGGAGC
LRRC19	HGLibB_27234	4	+	26995666	26995685	CACCGTCCAGTAAAACCATCTTCAT	AAACATGAAGATGGTTTTACTGGAC
LRRC19	HGLibB_27235	5	+	26996410	26996429	CACCGGAGTGGCATTCAGCCTTATG	AAACCATAAGGCTGAATGCCACTCC
LRRC19	HGLibB_27236	6	-	26996426	26996445	CACCGTATCAAAACAGTACCTCATA	AAACTATGAGGTACTGTTTTGATAC
MLLT3	HGLibA_29416	1	+	20448204	20448223	CACCGGTGGATGGCCTTCAAGATGC	AAACGCATCTTGAAGGCCATCCACC
MLLT3	HGLibA_29417	2	+	20448236	20448255	CACCGTAATCAAAGCGGACTTTCCT	AAACAGGAAAGTCCGCTTTGATTAC
MLLT3	HGLibA_29418	3	-	20622246	20622265	CACCGGCCCCCAGGCATGGCTAGCT	AAACAGCTAGCCATGCCTGGGGGCC
MLLT3	HGLibB_29375	4	-	20456751	20456770	CACCGCCTTACAAAGTAGAAGAATC	AAACGATTCTTCTACTTTGTAAGGC
MLLT3	HGLibB_29376	5	+	20456748	20456767	CACCGCCAGATTCTTCTACTTTGTA	AAACTACAAAGTAGAAGAATCTGGC
MLLT3	HGLibB_29377	6	-	20448215	20448234	CACCGGACTTATTCCTGCATCTTGA	AAACTCAAGATGCAGGAATAAGTCC
MOB3B	HGLibA_29596	1	-	27359140	27359159	CACCGTCTATATCCACCACTTCGAC	AAACGTCGAAGTGGTGGATATAGAC
MOB3B	HGLibA_29597	2	-	27359049	27359068	CACCGGATGAACCTCATAGACCGCA	AAACTGCGGTCTATGAGGTTCATCC
MOB3B	HGLibA_29598	3	+	27455121	27455140	CACCGATGAACTTACCCACGCATGT	AAACACATGCGTGGGTAAGTTCATC
MOB3B	HGLibB_29555	4	+	27455246	27455265	CACCGCTGCCACCGATACTCATATT	AAACAATATGAGTATCGGTGGCAGC
MOB3B	HGLibB_29556	5	-	27330600	27330619	CACCGTCCAGAAAGAAATGACGAGC	AAACGCTCGTCATTTCTTTCTGGAC
MOB3B	HGLibB_29557	6	-	27455495	27455514	CACCGACAAGACCTTCCGACCCAAG	AAACCTTGGGTCGGAAGGTCTTGTC
MTAP	HGLibA_30318	1	-	21837999	21838018	CACCGACTACACACCTCTCTCGTTT	AAACAAACGAGAGAGGTGTGTAGTC
MTAP	HGLibA_30319	2	+	21818069	21818088	CACCGTCAACTACCAGGCGAACATC	AAACGATGTTCGCCTGGTAGTTGAC
MTAP	HGLibA_30320	3	-	21816763	21816782	CACCGAAATACCATACCTTGCAAGG	AAACCCTTGCAAGGTATGGTATTTC
MTAP	HGLibB_30277	4	+	21818046	21818065	CACCGGCACACCATCATGCCTTCAA	AAACTTGAAGGCATGATGGTGTGCC
MTAP	HGLibB_30278	5	-	21837970	21837989	CACCGACGGCTCAGCCATTGGAATA	AAACTATTCCAATGGCTGAGCCGTC
MTAP	HGLibB_30279	6	-	21815510	21815529	CACCGTGGATATTAACCTTGCCAAA	AAACTTTGGCAAGGTTAATATCCAC
NDUFB6	HGLibA_31523	1	-	32573043	32573062	CACCGCGACATGACGGGGTACACTC	AAACGAGTGTACCCCGTCATGTCGC
NDUFB6	HGLibA_31524	2	-	32570994	32571013	CACCGCTCATGTACTTGTACCTGTC	AAACGACAGGTACAAGTACATGAGC

NDUFB6	HGLibA_31525	3	+	32573021	32573040	CACCGTGCAGCCGCAGTTTCTCATC	AAACGATGAGAAACTGCGGCTGCAC
NDUFB6	HGLibB_31481	4	-	32553930	32553949	CACCGTTCCTAGGGTGATACAATTC	AAACGAATTGTATCACCCTAGGAAC
NDUFB6	HGLibB_31482	5	+	32558899	32558918	CACCGAAGACTTACAGGGAATATTC	AAACGAATATTCCCTGTAAGTCTTC
NDUFB6	HGLibB_31483	6	-	32558942	32558961	CACCGTTATTACAGGAAAAACCATA	AAACTATGGTTTTTCCTGTAATAAC
NFX1	HGLibA_31847	1	+	33301310	33301329	CACCGCTGTGAATTGGTTCGTGTCA	AAACTGACACGAACCAATTCACAGC
NFX1	HGLibA_31848	2	-	33301407	33301426	CACCGAATTGACCTGCTTGAGATGC	AAACGCATCTCAAGCAGGTCAATTC
NFX1	HGLibA_31849	3	+	33307192	33307211	CACCGGCAAGGTAAAGAATCCTGAG	AAACCTCAGGATTCTTTACCTTGCC
NFX1	HGLibB_31804	4	+	33307221	33307240	CACCGAATGAAATTCCACATAGCTG	AAACCAGCTATGTGGAATTTCATTC
NFX1	HGLibB_31805	5	+	33311144	33311163	CACCGATGACAAAAACATGTGAATG	AAACCATTCACATGTTTTTGTCATC
NFX1	HGLibB_31806	6	+	33311096	33311115	CACCGTCTTTCAGTCTCTGCCATCC	AAACGGATGGCAGAGACTGAAAGAC
NOL6	HGLibA_32270	1	+	33472336	33472355	CACCGTTCAGCCAATGTACGCTTCC	AAACGGAAGCGTACATTGGCTGAAC
NOL6	HGLibA_32271	2	+	33472253	33472272	CACCGCGATTAAGCTCCTCATTGGT	AAACACCAATGAGGAGCTTAATCGC
NOL6	HGLibA_32272	3	+	33472019	33472038	CACCGAGGGCACCCTCACAACCCGC	AAACGCGGGTTGTGAGGGTGCCCTC
NOL6	HGLibB_32227	4	-	33469085	33469104	CACCGCTACCCCCCGCTATAACACA	AAACTGTGTTATAGCGGGGGGTAGC
NOL6	HGLibB_32228	5	+	33469057	33469076	CACCGACTCGAGAACTGTATCTTGC	AAACGCAAGATACAGTTCTCGAGTC
NOL6	HGLibB_32229	6	-	33472029	33472048	CACCGAGGTCAACCAGCGGGTTGTG	AAACCACAACCCGCTGGTTGACCTC
PLAA	HGLibA_36929	1	+	26928160	26928179	CACCGGTCAACATTAAGCCCTGTTC	AAACGAACAGGGCTTAATGTTGACC
PLAA	HGLibA_36930	2	-	26928176	26928195	CACCGGGTAAAGATCTTACCTGAAC	AAACGTTCAGGTAAGATCTTTACCC
PLAA	HGLibA_36931	3	-	26928339	26928358	CACCGGGGACACCACTGCTAAAGTC	AAACGACTTTAGCAGTGGTGTCCCC
PLAA	HGLibB_36882	4	-	26928308	26928327	CACCGCAAGTGCATGATGACCTTGC	AAACGCAAGGTCATCATGCACTTGC
PLAA	HGLibB_36883	5	-	26926441	26926460	CACCGGAGTGTCTTGAAGTATATTA	AAACTAATATACTTCAAGACACTCC
PLAA	HGLibB_36884	6	+	26926482	26926501	CACCGAATACTAGCATCATTTGCAC	AAACGTGCAAATGATGCTAGTATTC
PLIN2	HGLibB_37124	1	-	19126229	19126248	CACCGCTCAGCCTATCTCAGTACAA	AAACTTGTACTGAGATAGGCTGAGC
PLIN2	HGLibB_37125	2	+	19121128	19121147	CACCGTGCCCCAGTCACAGCGCCTT	AAACAAGGCGCTGTGACTGGGGCAC
PLIN2	HGLibB_37126	3	+	19123552	19123571	CACCGCCCAAGCTCACCTGAGTTGA	AAACTCAACTCAGGTGAGCTTGGGC
PLIN2	HGLibA_37172	4	+	19121059	19121078	CACCGCATCACCCCTGTGATCGTGC	AAACGCACGATCACAGGGGTGATGC
PLIN2	HGLibA_37173	5	-	19119725	19119744	CACCGTCACTCCCGTGCCTACCAGC	AAACGCTGGTAGGCACGGGAGTGAC
PLIN2	HGLibA_37174	6	-	19119650	19119669	CACCGGCTCCATTCTACTGTTCACC	AAACGGTGAACAGTAGAATGGAGCC
PRSS3	HGLibA_38922	1	+	33750672	33750691	CACCGGACGCACTTGGCGAGCGGCG	AAACCGCCGCTCGCCAAGTGCGTCC
PRSS3	HGLibA_38923	2	-	33797979	33797998	CACCGGGACACGCGGGCATTGATGA	AAACTCATCAATGCCCGCGTGTCCC
PRSS3	HGLibA_38924	3	-	33750662	33750681	CACCGCAAGTGCGTCCACCCAGAAG	AAACCTTCTGGGTGGACGCACTTGC
PRSS3	HGLibB_38873	4	-	33750841	33750860	CACCGGTCCGCGTACCCTCCCCCGC	AAACGCGGGGGAGGGTACGCGGACC
PRSS3	HGLibB_38874	5	-	33797918	33797937	CACCGAGAGTGTCCCTGTTGTATTT	AAACAAATACAACAGGGACACTCTC
PRSS3	HGLibB_38875	6	+	33794790	33794809	CACCGATGCACATGAGAGAGACAAG	AAACCTTGTCTCTCTCATGTGCATC
PTPLAD2	HGLibA_39420	1	+	21015967	21015986	CACCGGACTGGTGATCACCACAAAA	AAACTTTTGTGGTGATCACCAGTCC
PTPLAD2	HGLibA_39421	2	+	21011637	21011656	CACCGGAGCCATGTCAAGACAGCAT	AAACATGCTGTCTTGACATGGCTCC
PTPLAD2	HGLibA_39422	3	-	21011643	21011662	CACCGTATCCTATGCTGTCTTGACA	AAACTGTCAAGACAGCATAGGATAC
PTPLAD2	HGLibB_39371	4	-	21029350	21029369	CACCGTATTACTTAATCCAGTTCTG	AAACCAGAACTGGATTAAGTAATAC
PTPLAD2	HGLibB_39372	5	+	21015934	21015953	CACCGCACACACCACATATTTCTCT	AAACAGAGAAATATGTGGTGTGTGC
PTPLAD2	HGLibB_39373	6	+	21029324	21029343	CACCGTATTTGTAAATATCCAAGAG	AAACCTCTTGGATATTTACAAATAC
RPS6	HGLibA_42154	1	-	19378465	19378484	CACCGTACTACAGTGCCTCGCCGCC	AAACGGCGGCGAGGCACTGTAGTAC

RPS6	HGLibA_42155	2	+	19376483	19376502	CACCGTAGACTAACCTTCATTCTCT	AAACAGAGAATGAAGGTTAGTCTAC
RPS6	HGLibA_42156	3	+	19378358	19378377	CACCGCCTCCTACCTTCTTTATTTA	AAACTAAATAAAGAAGGTAGGAGGC
RPS6	HGLibB_42101	4	-	19378724	19378743	CACCGAAATCTGAGCGTTCTCAACT	AAACAGTTGAGAACGCTCAGATTTC
RPS6	HGLibB_42102	5	+	19376572	19376591	CACCGTACGCCGCCGTTTGTGCTGC	AAACGCAGCACAAACGGCGGCGTAC
RPS6	HGLibB_42103	6	-	19379529	19379548	CACCGTACTTTCTATGAGAAGCGTA	AAACTACGCTTCTCATAGAAAGTAC
RRAGA	HGLibB_42170	1	-	19049746	19049765	CACCGGGTGTCGCGAGCAATGTAAT	AAACATTACATTGCTCGCGACACCC
RRAGA	HGLibB_42171	2	+	19049891	19049910	CACCGAGACAATATCTTCCGTAACG	AAACCGTTACGGAAGATATTGTCTC
RRAGA	HGLibB_42172	3	+	19049966	19049985	CACCGGCATTATTACCAGTCGTGTC	AAACGACACGACTGGTAATAATGCC
RRAGA	HGLibA_42223	4	+	19049746	19049765	CACCGATTACATTGCTCGCGACACC	AAACGGTGTCGCGAGCAATGTAATC
RRAGA	HGLibA_42224	5	+	19050455	19050474	CACCGCACCTCAAATACGTACGTGA	AAACTCACGTACGTATTTGAGGTGC
RRAGA	HGLibA_42225	6	-	19049978	19049997	CACCGGGATGGCCTCCAGACACGAC	AAACGTCGTGTCTGGAGGCCATCCC
SH3GL2	HGLibA_43948	1	-	17795563	17795582	CACCGGTCGTACAGAGCTCGGCAGC	AAACGCTGCCGAGCTCTGTACGACC
SH3GL2	HGLibA_43949	2	-	17795732	17795751	CACCGGCATAACATCCTAATGGGGC	AAACGCCCCATTAGGATGTTATGCC
SH3GL2	HGLibA_43950	3	+	17793404	17793423	CACCGACCTAAACCACGAATGAGCC	AAACGGCTCATTCGTGGTTTAGGTC
SH3GL2	HGLibB_43895	4	-	17791303	17791322	CACCGGTCTGACCGTGACTTGCTGC	AAACGCAGCAAGTCACGGTCAGACC
SH3GL2	HGLibB_43896	5	-	17793390	17793409	CACCGTTAGGTTGATATTCCCTTCT	AAACAGAAGGGAATATCAACCTAAC
SH3GL2	HGLibB_43897	6	+	17791252	17791271	CACCGTGCACTTGTGCAAGCTCAGC	AAACGCTGAGCTTGCACAAGTGCAC
SLC24A2	HGLibB_44564	1	-	19786746	19786765	CACCGCTGAAGTTAATTCGAGTCTT	AAACAAGACTCGAATTAACTTCAGC
SLC24A2	HGLibB_44565	2	-	19622261	19622280	CACCGCAAGGATGAACCAACTCTAC	AAACGTAGAGTTGGTTCATCCTTGC
SLC24A2	HGLibB_44566	3	+	19622248	19622267	CACCGCTGCTTCCTACCGGTAGAGT	AAACACTCTACCGGTAGGAAGCAGC
SLC24A2	HGLibA_44617	4	+	19619572	19619591	CACCGTGATGTTTACCTTCGGCGAG	AAACCTCGCCGAAGGTAAACATCAC
SLC24A2	HGLibA_44618	5	-	19619584	19619603	CACCGACCCTTGACCCACTCGCCGA	AAACTCGGCGAGTGGGTCAAGGGTC
SLC24A2	HGLibA_44619	6	+	19785958	19785977	CACCGTCACCTTGACGACCTTATTG	AAACCAATAAGGTCGTCAAGGTGAC
SMU1	HGLibA_45838	1	-	33073596	33073615	CACCGCCTCATTGACCTCTATGAAC	AAACGTTCATAGAGGTCAATGAGGC
SMU1	HGLibA_45839	2	+	33068858	33068877	CACCGCACACTGACTTCGCCAGCTA	AAACTAGCTGGCGAAGTCAGTGTGC
SMU1	HGLibA_45840	3	+	33073584	33073603	CACCGCCACTCTTACCTGTTCATAG	AAACCTATGAACAGGTAAGAGTGGC
SMU1	HGLibB_45783	4	+	33062107	33062126	CACCGCTTGCCTCGAAACAAATCTA	AAACTAGATTTGTTTCGAGGCAAGC
SMU1	HGLibB_45784	5	-	33068860	33068879	CACCGCTTAGCTGGCGAAGTCAGTG	AAACCACTGACTTCGCCAGCTAAGC
SMU1	HGLibB_45785	6	-	33062114	33062133	CACCGATGACCATAGATTTGTTTCG	AAACCGAAACAAATCTATGGTCATC
SPINK4	HGLibA_46693	1	+	33240202	33240221	CACCGTCAGCATGGCCGTCCGCCAG	AAACCTGGCGGACGGCCATGCTGAC
SPINK4	HGLibA_46694	2	-	33240221	33240240	CACCGAGGCCAGGGCGATTACCCAC	AAACGTGGGTAATCGCCCTGGCCTC
SPINK4	HGLibA_46695	3	-	33246667	33246686	CACCGGCCCATCAGTGCCGCAGACC	AAACGGTCTGCGGCACTGATGGGCC
SPINK4	HGLibB_46636	4	-	33246644	33246663	CACCGTTGGACATCTGGGAACAGGT	AAACACCTGTTCCCAGATGTCCAAC
SPINK4	HGLibB_46637	5	-	33248445	33248464	CACCGATTTGCCATCTTTCATGATC	AAACGATCATGAAAGATGGCAAATC
SPINK4	HGLibB_46638	6	-	33245139	33245158	CACCGTGCCTTACCATTCTTGAGAA	AAACTTCTCAAGAATGGTAAGGCAC
TAF1L	HGLibA_48224	1	-	32632728	32632747	CACCGATGAAGTTCACGCTGCTCCT	AAACAGGAGCAGCGTGAACTTCATC
TAF1L	HGLibA_48225	2	-	32634276	32634295	CACCGATTGGGAGGATTCTATCATC	AAACGATGATAGAATCCTCCCAATC
TAF1L	HGLibA_48226	3	-	32635172	32635191	CACCGCTTGCAGCCCCTTTACCACT	AAACAGTGGTAAAGGGGCTGCAAGC
TAF1L	HGLibB_48165	4	-	32634448	32634467	CACCGATGCTGGGTGTCTCCGAAGA	AAACTCTTCGGAGACACCCAGCATC
TAF1L	HGLibB_48166	5	+	32635154	32635173	CACCGCATCTTCATCATAATCCGAG	AAACCTCGGATTATGATGAAGATGC
TAF1L	HGLibB_48167	6	+	32634773	32634792	CACCGTCCCTGGTCCAAAAAGATGT	AAACACATCTTTTTGGACCAGGGAC

TEK	HGLibA_48967	1	+	27202912	27202931	CACCGTTCTATTACTATCCGTTACA	AAACTGTAACGGATAGTAATAGAAC
TEK	HGLibA_48968	2	+	27197355	27197374	CACCGAGACCACTCTAAATTTGACC	AAACGGTCAAATTTAGAGTGGTCTC
TEK	HGLibA_48969	3	+	27197527	27197546	CACCGCCGAGCTAGAGTCAACACCA	AAACTGGTGTTGACTCTAGCTCGGC
TEK	HGLibB_48908	4	-	27202989	27203008	CACCGCTTGAGCTGATACTGAGTGA	AAACTCACTCAGTATCAGCTCAAGC
TEK	HGLibB_48909	5	-	27173306	27173325	CACCGCAGGAACACCCATAGGGGTC	AAACGACCCCTATGGGTGTTCCTGC
TEK	HGLibB_48910	6	+	27173246	27173265	CACCGACTTGTAAAGAAAGGTGCAG	AAACCTGCACCTTTCTTTACAAGTC
TOPORS	HGLibA_51011	1	-	32784784	32784803	CACCGGTCTCCGCTGTCTCGCGAGG	AAACCCTCGCGAGACAGCGGAGACC
TOPORS	HGLibA_51012	2	-	32784874	32784893	CACCGAGGATCCGCGAAGGCGTACC	AAACGGTACGCCTTCGCGGATCCTC
TOPORS	HGLibA_51013	3	+	32784186	32784205	CACCGGCTACCGTACAACTCTGACA	AAACTGTCAGAGTTGTACGGTAGCC
TOPORS	HGLibB_50948	4	+	32784230	32784249	CACCGCTGCATCTACCGGGACTGAC	AAACGTCAGTCCCGGTAGATGCAGC
TOPORS	HGLibB_50949	5	+	32784470	32784489	CACCGCCGACACCGACCTAGCTTTC	AAACGAAAGCTAGGTCGGTGTCGGC
TOPORS	HGLibB_50950	6	-	32784660	32784679	CACCGGATCTTTCATCTGCCGTAGT	AAACACTACGGCAGATGAAAGATCC
TMEM215	HGLibA_50218	1	-	32550927	32550946	CACCGAGCAAACGATGATACTGTCC	AAACGGACAGTATCATCGTTTGCTC
TMEM215	HGLibA_50219	2	+	32550852	32550871	CACCGGAGATTAGACGATGGTCTCG	AAACCGAGACCATCGTCTAATCTCC
TMEM215	HGLibA_50220	3	-	32543968	32543987	CACCGGGCCTGATGACATTAACCCG	AAACCGGGTTAATGTCATCAGGCCC
TMEM215	HGLibB_50158	4	-	32542168	32542187	CACCGGTCAGTGTCTTCCTCGTCTT	AAACAAGACGAGGAAGACACTGACC
TMEM215	HGLibB_50159	5	-	32550831	32550850	CACCGACCCCTTCAGACCTAGAATC	AAACGATTCTAGGTCTGAAGGGGTC
TMEM215	HGLibB_50160	6	+	32543786	32543805	CACCGGCAGTAGCCGTCCAGGTATC	AAACGATACCTGGACGGCTACTGCC
TUSC1	HGLibA_52341	1	+	25678266	25678285	CACCGGCAGCAACTCCCGCGCCTAG	AAACCTAGGCGCGGGAGTTGCTGCC
TUSC1	HGLibA_52342	2	+	25678074	25678093	CACCGCCACTCGTCCCGCGCACGGA	AAACTCCGTGCGCGGGACGAGTGGC
TUSC1	HGLibA_52343	3	-	25677727	25677746	CACCGCCTCTACAGGAACCCGACTC	AAACGAGTCGGGTTCCTGTAGAGGC
TUSC1	HGLibB_52276	4	+	25678097	25678116	CACCGCCTCCAAGTGGCTCGCCGCC	AAACGGCGGCGAGCCACTTGGAGGC
TUSC1	HGLibB_52277	5	-	25678284	25678303	CACCGGCATGCGTGGTGGCGCCACT	AAACAGTGGCGCCACCACGCATGCC
TUSC1	HGLibB_52278	6	-	25677958	25677977	CACCGTTGCGGCTCCCCGGCGAAGG	AAACCCTTCGCCGGGGAGCCGCAAC
UBAP2	HGLibA_52551	1	+	34017101	34017120	CACCGTTTCCCGAGCACCTCGACAA	AAACTTGTCGAGGTGCTCGGGAAAC
UBAP2	HGLibA_52552	2	+	33927919	33927938	CACCGTGAGACGGAGGTCGCTGCCG	AAACCGGCAGCGACCTCCGTCTCAC
UBAP2	HGLibA_52553	3	+	33927868	33927887	CACCGGTTCGCGGTGTTCATGCTAC	AAACGTAGCATGAACACCGCGAACC
UBAP2	HGLibB_52486	4	-	34017115	34017134	CACCGGTGAGCAGTGACCATTGTCG	AAACCGACAATGGTCACTGCTCACC
UBAP2	HGLibB_52487	5	-	33927905	33927924	CACCGGTCTCAAGTTCCGCATCCTC	AAACGAGGATGCGGAACTTGAGACC
UBAP2	HGLibB_52488	6	-	33927797	33927816	CACCGGCCGCGCCCTTGGTGACCTC	AAACGAGGTCACCAAGGGCGCGGCC
UBE2R2	HGLibA_52653	1	-	33912021	33912040	CACCGCATAACTGAAGCATCGACAT	AAACATGTCGATGCTTCAGTTATGC
UBE2R2	HGLibA_52654	2	-	33886897	33886916	CACCGGGTGAATAGGGGTAGTCAAT	AAACATTGACTACCCCTATTCACCC
UBE2R2	HGLibA_52655	3	+	33900248	33900267	CACCGGGAATCCTACTCAGAATGTG	AAACCACATTCTGAGTAGGATTCCC
UBE2R2	HGLibB_52588	4	-	33911971	33911990	CACCGGCAGTGAGATTACACTTAAT	AAACATTAAGTGTAATCTCACTGCC
UBE2R2	HGLibB_52589	5	-	33900256	33900275	CACCGCCTTACCTCACATTCTGAGT	AAACACTCAGAATGTGAGGTAAGGC
UBE2R2	HGLibB_52590	6	-	33886955	33886974	CACCGTCTCCTTACCTCATAAATGT	AAACACATTTATGAGGTAAGGAGAC

Supplementary Table 4

Hemizygous deletion frequencies. Number (*N*) and frequency (%) of hemizygous/incomplete deletions of identified genes in TCGA, CCLE and Tumorscape.

				TCGA		CCLE		Tumorscape	
**GeneSymbol**	**Chr**	**Start**	**End**	***N***	**%**	***N***	**%**	***N***	**%**
**4q34.3 *LINC00290***									
LOC90768 *	4	183060158	183065668	799	10.9	254	24.4	201	8.1
MIR1305 *	4	183090445	183090531	797	10.9	255	24.4	200	8.1
DCTD	4	183811243	183838630	793	10.8	256	24.5	197	8.0
FAM92A1P2 *	4	183958817	183961272	794	10.9	248	23.8	204	8.2
**9p21.3 *CDKN2A***									
FAM154A	9	18927890	19033256	1005	13.7	300	28.8	273	11.0
RRAGA	9	19049371	19051021	1000	13.7	298	28.6	269	10.9
HAUS6	9	19053134	19102940	1002	13.7	298	28.6	270	10.9
SCARNA8 *	9	19063653	19063784	999	13.7	298	28.6	269	10.9
PLIN2	9	19115758	19127604	995	13.6	299	28.7	269	10.9
DENND4C	9	19230762	19374137	1012	13.8	301	28.9	274	11.1
RPS6	9	19376253	19380235	995	13.6	297	28.5	273	11.0
ACO1	9	32384600	32450832	761	10.4	174	16.7	201	8.1
DDX58	9	32455299	32526322	746	10.2	173	16.6	198	8.0
**10q23.31 *PTEN***									
WAPAL	10	88195012	88281541	963	13.2	161	15.4	171	6.9
FAM22A	10	88985204	88994733	854	11.7	152	14.6	149	6.0
FAM22A-AS1 *	10	88998423	89102315	1111	15.2	196	18.8	149	6.0
LOC439994	10	89102167	89103331	1056	14.4	186	17.8	149	6.0
FAM22D	10	89117476	89130452	919	12.6	168	16.1	151	6.1
HTR7	10	92500575	92617671	990	13.5	158	15.1	180	7.3
RPP30	10	92631708	92668312	982	13.4	158	15.1	177	7.1
ANKRD1	10	92671856	92681032	985	13.5	159	15.2	177	7.1
**13q14.2 *RB1***									
SUCLA2	13	48516790	48575462	973	13.3	245	23.5	257	10.4
NUDT15	13	48611702	48621282	970	13.3	246	23.6	256	10.3
MED4	13	48649863	48669277	973	13.3	245	23.5	256	10.3
MED4-AS1 *	13	48651272	48654129	972	13.3	245	23.5	256	10.3
**17p12 *MAP2K4***									
MYH13	17	10204182	10276322	1321	18.1	260	24.9	310	12.5
MYH8	17	10293641	10325267	1323	18.1	260	24.9	308	12.4
MYH4	17	10346607	10372876	1315	18.0	261	25.0	307	12.4
MYH1	17	10395626	10421859	1323	18.1	261	25.0	307	12.4
MYH2	17	10424464	10452940	1324	18.1	262	25.1	307	12.4

MYH3	17	10531842	10560626	1319	18.0	258	24.7	311	12.6
SCO1	17	10583648	10600885	1318	18.0	258	24.7	312	12.6
ELAC2	17	12894928	12921381	1331	18.2	249	23.9	304	12.3
**17q11.2 *NF1***									
GOSR1	17	28804425	28853832	584	8.0	91	8.7	91	3.7
SUZ12P1	17	29036625	29097068	579	7.9	92	8.8	89	3.6
CRLF3	17	29109701	29151778	600	8.2	91	8.7	88	3.6
ATAD5	17	29159022	29222295	614	8.4	98	9.4	89	3.6
TEFM	17	29226000	29233286	604	8.3	98	9.4	87	3.5
ADAP2	17	29248753	29286211	610	8.3	99	9.5	88	3.6
COPRS	17	30178883	30186326	619	8.5	97	9.3	84	3.4
LRRC37B	17	30348154	30380519	615	8.4	93	8.9	82	3.3
*SH3GL1P1 **	17	30367354	30369851	612	8.4	93	8.9	82	3.3
RHOT1	17	30469472	30552746	615	8.4	89	8.5	87	3.5
*ARGFXP2 **	17	30477386	30478590	613	8.4	89	8.5	85	3.4
**18q21.2 *SMAD4***									
CCDC11	18	47753562	47792865	972	13.3	341	32.7	189	7.6
MBD1	18	47793251	47808144	972	13.3	341	32.7	189	7.6
CXXC1	18	47808712	47814692	971	13.3	341	32.7	187	7.6
SKA1	18	47901391	47920538	974	13.3	340	32.6	186	7.5

** non protein coding gene*

Supplementary Table 5

PCR primer sequences used for detecting CRISPR-Induced Insertion/Deletions.

gene_id	UID	sgRNA nr	Forward primer (5'-3')	Reverse primer (5'-3')
ACER2	HGLibB_00430, HGLibB_00431	1,2	CCAATGCCCCGGAGTG	AGAGACCCACGTCCAGCTT
ACER2	HGLibB_00432	3,6	TTTGCCCTTTTTACTTAATACTCATC	AATTCCTTCTACCCCCATCC
ACER2	HGLibB_00430, HGLibB_00431	4,5	TTGAATTTGTGTCTCTTCTGTGG	AAGATCTTTGGTAGATACCTTCTGG
ACO1	HGLibA_00460	1	GGGTCAGCCAATCAGTATGG	TGGAAACCAAACAAGATTCCA
ACO1	HGLibA_00461	2	TTTATATAGAGATTGGCCTTAAAGA	TTCTTGTGCTGCGTGACATT
ACO1	HGLibA_00462	3	GATTTCTAGGTCCCAGACCTCTT	TCACAGCCATGTACCTACCA
ACO1	HGLibB_00460	4	CCATTCGGAATTGTGATGAG	AAAACTTTCTTGAAAGGCTAATGA
ACO1	HGLibB_00461, HGLibB_00462	5,6	GCTGTGGTTGACTTTGCTG	AAAGATTTTGCACAAAGCAGAA
ANKRD18B	HGLibA_02020, HGLibA_02021	1,2	GTGGAAAGGCCACGAGGA	CAGGCAGTGCTCCACCTC
ANKRD18B	HGLibA_02022, HGLibA_02019	3,5	CCTGTGGAATATGTATTTTGAATTCTT	TCTGTACCTTGTTTAGTGCTTCAA
ANKRD18B	HGLibB_02018	4	GACTGTTCTACATTTGGCCTGT	CCACAGGTTTCACCAACTAGC
AQP3	HGLibA_02592, HGLibA_02593	1,2	TCCTATAAAGGGAGCCACCA	CCCTCCACTCACCACCAG
AQP3	HGLibA_02594, HGLibA_02591	3,5	GTGGCAGGCTGCAGCTAATA	GGTGGGGAATGCTTACCATA
AQP3	HGLibB_02590	4	GAGAGTTGCTGGTCCTCACC	AAGGCCTTACCAGAGACCTG
AQP3	HGLibB_02592	6	TCTAATCTGTCACCAGATGCAA	CCATGAGCTACCAAGGCAAC
AQP7	HGLibA_02604, HGLibA_02605	1,2	CTGTTGTTTGTTCTGCTCTCACT	CTGTAGAAGAGACTGTAGATGGTG
AQP7	HGLibA_02606, HGLibA_02603	3,5	TCAGCTGGGAGTTGAAGAGC	ACCCACTCACCATCATGACA
AQP7	HGLibB_02602, HGLibB_02604	4,6	TTGTAGGTATTCGGCCTTGG	GGGCAGGAAAGAGCCTGT
B4GALT1	HGLibA_04022, HGLibA_04024	1,3	CAATGTTGGCTTTCAAGAAG	CTAGTCAACACATGAGAGAGACAT
B4GALT1	HGLibA_04023	2	GCAAAGCAGAACCCAAATGT	AGGCCTCACCTGGTTGATAA
B4GALT1	HGLibB_04020	4	ATCTGATGCAGCCTGTGGA	GCCACCTTGTGAGGAGAGAC
B4GALT1	HGLibB_04021	5	GGAGACAATGCTCTCTGATGG	GACCAGCCCAGCAGATTG
B4GALT1	HGLibB_04022	6	GGGAGGAGGAAGTACGTGTG	TGTGATTTGGGTATACAATGGGTA
DENND4C	HGLibB_12937	1	CATTGCCATTATTATCAAATCAGC	CCAATATCTGTAAGCGGTGGT
DENND4C	HGLibB_12938, HGLibB_12939	2,3	GTTGGTATCCAACAAAATCAA	TCTTTTAGCCAAATGTTAATATGC

DENND4C	HGLibA_12955	4	GGTATCTTTCCCCTCCCTCTT	TGGAGCTTTAGGTCCAGTTGA
DENND4C	HGLibA_12956, HGLibA_12957	5,6	TCTATTTCTTCCCCTTCCTG	CAGTTACAGCCAAGGAATTCTG
DNAJA1	HGLibA_13509, HGLibA_13510	1,2	TGATTCGTGATTTGAAAAATATAAA	TACTACACTTTGTTAACAGGAGACA
DNAJA1	HGLibA_13511	3	GCATTTAGGAATTGTCTTCTTAAAAC	GCACTCCATGCACACAGACT
DNAJA1	HGLibB_13490	4	GGAGCGGATCAGTCCTAAAG	TGTTAATAATAAATCTGCCACAGCA
DNAJA1	HGLibB_13491	5	TGAAATTCACTCCTCTTTTCTCAA	ATCCTTCCTCCTCCTCCAAA
DNAJA1	HGLibB_13492	6	CCAGCATCTTAAACAGATTTGC	TTCAGGTGTCATATTAAAGGTGCT
FAM154A	HGLibB_16151	1	GACTGCCTGACCACCACTC	CCAGGAGGCTCAGGATATGA
FAM154A	HGLibB_16152, HGLibB_16153	2,3	AAACCATGTCTTCTCTCCGAAT	CATTAGCACTGCATGTTACTCC
FAM154A	HGLibA_16173, HGLibA_16174	4,5	TTGCTCACGACGTATAAGAAAGA	TGCACATAAAACACATTTCCTG
FAM154A	HGLibA_16175	6	CCACTTCGTCTGGAACACAA	TGGGCCACATAGCTCATCTT
HAUS6	HGLibB_20860, HGLibB_20861	1,2	TGTGATGTAAATGTAAGAGTTATTGTT	TGAACTTACTTTTAGAATTGGATTTA
HAUS6	HGLibB_20862, HGLibB_20888, HGLibB_20889	3,4,5	GCCTGTTTTCTATAATCTTATTTGTG	GGTGACATCTGTTCAAAAGAAA
HAUS6	HGLibA_20890	6	TGTGCATAGTCCTTACGCTTTT	AAGCATTAAAAGGATTTCACCTC
NDUFB6	HGLibA_31523, HGLibA_31525	1,3	GGGAGCGCGCTTAAGTAAC	TAGGCCCCATCTTCTGTGG
NDUFB6	HGLibA_31524	2	TGGGATATGTTTATTTTGTGTATGTG	GCCACAAAGAAAGTAATTTTGGA
NDUFB6	HGLibB_31481	4	TCCATGTGAGATCTGATGAACA	GGCTCATAAGCCTTTTAACTTTTT
NDUFB6	HGLibB_31482, HGLibB_31483	5,6	AAAATTTGGGGTATGACCTATTT	AATCTAATAATTGCTTGGAAAGG
NOL6	HGLibA_32270, HGLibA_32271	1,2	TCACTGGCCACAGGTGAT	AGCTGCAGACACTCAACAGT
NOL6	HGLibA_32272, HGLibA_32229	3,6	CAGGTAGAGGAGCTACTAAAGGA	GGCCCCTGTTTGATATACCC
NOL6	HGLibB_32227, HGLibB_32228	4,5	AGCTGGAGGGAAGAGGTGT	CAGAAGTGCCACGCCATC
PLIN2	HGLibB_37124	1	CTTTGATTGCAGAGTGTGGTG	CTTCTGGATGATGGGCAGAG
PLIN2	HGLibB_37125, HGLibB_37172	2,4	TGTCCTTGTGCTCATTTTTC	TGTTAATGCTGCCACTGACC
PLIN2	HGLibB_37126	3	GCAGTTGCCAATACCTATGC	GATCCAGGTTGGGAAAACAA
PLIN2	HGLibA_37173, HGLibA_37174	5,6	AAGTTGAAGGATTTGATCTGGTTC	GCCTAGAGATAAATGAACCCACTT
PRSS3	HGLibA_38922, HGLibA_38924	1,3	CAAGTGTGGAAAGGGTCTGG	AGCCCTCCTCCAGTTTCAAG
PRSS3	HGLibA_38923, HGLibA_38874	2,5	ACAACATCAAAGTCCTGGAG	CCACCAAAGCTCAGAGTGT
PRSS3	HGLibB_38873	4	GGGCTTGAAACTGGAGGA	CTCCCGTCCTCCCCTAGA
PRSS3	HGLibB_38875	6	CCCTGAGCAGGTGAGTCAGT	GGAGGGGACACAGGTTAGGT
RPS6	HGLibA_42154	1	AGGATTCTTAAGGAAGTTGTTGAA	CAACATACTGGCGGACATCA
RPS6	HGLibA_42155	2	CGGCGTATTGCTCTGAAGAA	GCCTCCTAAACAAAACAAAACA

RPS6	HGLibA_42156	3	CCCCAAAAGAGCTAGCAGAA	AGTGCTAAGGCCTTCCAAAG
RPS6	HGLibB_42101	4	CCGCCTGCTACTGAGTAAGG	GCATTTGGACAACTGGCTTT
RPS6	HGLibB_42102	5	TGAAATGATTGAGTCCTTTGAATTT	TCTTGGCCAAAAGTTTAGCAT
RPS6	HGLibB_42103	6	CCTTAGCTGAACATCTCCTTCC	CAATGGCGTTTACCGTCTCT
RRAGA	HGLibB_42170, HGLibB_42223	1,4	TGCCAAATACAGCCATGAAG	CACAGGTTCAGCACCAGGTT
RRAGA	HGLibB_42171, HGLibB_42172, HGLibB_42225	2,3,6	CTGGTGCTGAACCTGTGG	CGCTGATCCTCCTGAACC
RRAGA	HGLibA_42224	5	AGTAAATTGGCCGCTTCCTT	CCATCCACTCTCTCCAGCTT
SMU1	HGLibA_45838, HGLibA_45840	1,3	TGTGGCTGACATTAACAGTGG	TGAAGGAATGCTCACTGACG
SMU1	HGLibA_45839, HGLibA_45784	2,5	ACACCCTCTCCTTTTTCAGG	CTCAGCCTCCCAAAGTCAAG
SMU1	HGLibB_45783, HGLibB_45785	4,6	AAGCACCTGAACAGATTGCTATATT	CTGACTGGCTGAATGTCTTAGG
TOPORS	HGLibA_51011, HGLibA_51012, HGLibA_50949	1,2,5	GCTCTCTGCCCTGCTTCC	TTGTTCCGAATCTCACTCACC
TOPORS	HGLibA_51013	3	CCATTCTGTGAGGGCAGAAG	CCACTATCCGGTGGAGTTGT
TOPORS	HGLibB_50948	4	TCTGAAAGAACAAATGCTAGGAAA	TGAGCCACTTCGTTAGTACCC
TOPORS	HGLibB_50950	6	TACTGTTTGAAGGGTTAGGCATT	TTCTAACTCGAGCACCAGCA
