# Supplementary material for: Essential genes shape cancer genomes through linear limitation of homozygous deletions
Source: Commun Biol. 2019 Jul 19;2:262. doi: 10.1038/s42003-019-0517-0 (PMC6642121; doi:10.1038/s42003-019-0517-0)
Supplement: Supplementary file 2 — Reporting Summary [file 42003_2019_517_MOESM2_ESM.pdf]

nature research

Corresponding author(s): Björn Nilsson 14-06-2019

Reporting Summary

Nature Research wishes to improve the reproducibility of the work that we publish. This form provides structure for consistency and transparency in reporting. For further information on Nature Research policies, see Authors & Referees and the Editorial Policy Checklist.

Statistical parameters

When statistical analyses are reported, confirm that the following items are present in the relevant location (e.g. figure legend, table legend, main text, or Methods section).

n/a	Confirmed	
☐	☒	The exact sample size (*n*) for each experimental group/condition, given as a discrete number and unit of measurement
☐	☒	An indication of whether measurements were taken from distinct samples or whether the same sample was measured repeatedly
☒	☐	The statistical test(s) used AND whether they are one- or two-sided<br>*Only common tests should be described solely by name; describe more complex techniques in the Methods section.*
☒	☐	A description of all covariates tested
☒	☐	A description of any assumptions or corrections, such as tests of normality and adjustment for multiple comparisons
☐	☒	A full description of the statistics including central tendency (e.g. means) or other basic estimates (e.g. regression coefficient) AND variation (e.g. standard deviation) or associated estimates of uncertainty (e.g. confidence intervals)
☒	☐	For null hypothesis testing, the test statistic (e.g. *F*, *t*, *r*) with confidence intervals, effect sizes, degrees of freedom and *P* value noted<br>*Give P values as exact values whenever suitable.*
☒	☐	For Bayesian analysis, information on the choice of priors and Markov chain Monte Carlo settings
☒	☐	For hierarchical and complex designs, identification of the appropriate level for tests and full reporting of outcomes
☒	☐	Estimates of effect sizes (e.g. Cohen's *d*, Pearson's *r*), indicating how they were calculated
☒	☐	Clearly defined error bars<br>*State explicitly what error bars represent (e.g. SD, SE, CI)*

Our web collection on statistics for biologists may be useful.

Software and code

Policy information about availability of computer code

Data collection	No software was used for data collection.
Data analysis	The analysis was done using various Winsdows and Linux scripts and Matlab.

For manuscripts utilizing custom algorithms or software that are central to the research but not yet described in published literature, software must be made available to editors/reviewers upon request. We strongly encourage code deposition in a community repository (e.g. GitHub). See the Nature Research guidelines for submitting code & software for further information.

Data

Policy information about availability of data

All manuscripts must include a data availability statement. This statement should provide the following information, where applicable:

- Accession codes, unique identifiers, or web links for publicly available datasets
- A list of figures that have associated raw data
- A description of any restrictions on data availability

We obtained segmented copy number (CN) data for 7,268 primary tumor samples belonging to 24 cancer types from The Cancer Genome Atlas (TCGA) (http://cancergenome.nih.gov/), 2,476 samples belonging to 39 cancer types from the Tumorscape compendium (http://www.broadinstitute.org/tumorscape) and 1,043 samples representing human cancer cell lines from the Cancer Cell Line Encyclopedia (https://portals.broadinstitute.org/ccle).

DepMap gene dependency scores were obtained from https://depmap.org/portal/download/all/ (DepMap Public 19Q1 release). The remaining data are contained within the paper and Supplementary Files or are available from the authors upon request.

Field-specific reporting

Please select the best fit for your research. If you are not sure, read the appropriate sections before making your selection.

☒ Life sciences ☐ Behavioural & social sciences ☐ Ecological, evolutionary & environmental sciences

For a reference copy of the document with all sections, see nature.com/authors/policies/ReportingSummary-flat.pdf

Life sciences study design

All studies must disclose on these points even when the disclosure is negative.

Sample size	We obtained segmented copy number (CN) data for 7,268 primary tumor samples belonging to 24 cancer types from The Cancer Genome Atlas (TCGA) (http://cancergenome.nih.gov/), 2,476 samples belonging to 39 cancer types from the Tumorscape compendium (http://www.broadinstitute.org/tumorscape), and 1,043 samples representing human cancer cell lines from the Cancer Cell Line Encyclopedia (https://portals.broadinstitute.org/ccle). No sample-size calculation was performed.
Data exclusions	No data were excluded from this study.
Replication	All lentiviral cell transductions were performed in triplicates with reproducible results.
Randomization	No experimental groups were formed and randomization was not relevant to this study.
Blinding	No experimental groups were formed and blinding was not relevant to this study.

Reporting for specific materials, systems and methods

Materials & experimental systems

n/a	Involved in the study
☒	☐ Unique biological materials
☒	☐ Antibodies
☐	☒ Eukaryotic cell lines
☒	☐ Palaeontology
☒	☐ Animals and other organisms
☒	☐ Human research participants

Methods

n/a	Involved in the study
☒	☐ ChIP-seq
☒	☐ Flow cytometry
☒	☐ MRI-based neuroimaging

Eukaryotic cell lines

Policy information about cell lines

Cell line source(s)	RCH-ACV (ACC 548, DSMZ), Jurkat (ACC 282, DSMZ)
Authentication	Both cell lines were acquired from DSMZ for the purposes of this study. No further authentication was performed.
Mycoplasma contamination	Both cell lines were acquired from DSMZ for the purposes of this study and were not tested for mycoplasma contamination.
Commonly misidentified lines (See ICLAC register)	No misidentified lines were used in the present study
